# Effective-component compatibility of Bufei Yishen formula III ameliorated COPD by improving airway epithelial cell senescence by promoting mitophagy via the NRF2/PINK1 pathway

**DOI:** 10.1186/s12890-022-02191-9

**Published:** 2022-11-22

**Authors:** Min-yan Li, Yan-qin Qin, Yan-ge Tian, Kang-chen Li, Brian G. Oliver, Xue-fang Liu, Peng Zhao, Jian-sheng Li

**Affiliations:** 1 Henan Key Laboratory of Chinese Medicine for Respiratory Disease, Henan University of Chinese Medicine, 156 Jinshui Dong road, Zhengzhou, 450046 Henan China; 2 Collaborative Innovation Center for Chinese Medicine and Respiratory Diseases co-constructed by Henan province & Education Ministry of P.R. China, Zhengzhou, 450046 Henan China; 3 Academy of Chinese Medical Sciences, Henan University of Chinese Medicine, Zhengzhou, 450046 Henan China; 4 School of Life Sciences, Faculty of Science, University of Technology Sydney, Sydney, New South Wales 2007 Australia; 5 Woolcock Institute of Medical Research, Respiratory Cellular and Molecular Biology, The University of Sydney, Sydney, New South Wales 2037 Australia; 6 Department of Respiratory Diseases, the First Affiliated Hospital of Henan University of Chinese Medicine, Zhengzhou, 450000 China

**Keywords:** Chronic obstructive pulmonary disease, Effective-component compatibility of Bufei Yishen formula III, Airway epithelial cell senescence, Oxidative stress, Mitophagy

## Abstract

**Background:**

Effective-component compatibility of Bufei Yishen formula III (ECC-BYF III) demonstrates positive effects on stable chronic obstructive pulmonary disease (COPD).

**Purpose:**

To investigate the mechanisms of ECC-BYF III on COPD rats from the aspect of airway epithelial cell senescence.

**Methods:**

COPD model rats (Sprague-Dawley rat) were treated with ECC-BYF III for 8 weeks, and the efficacy was evaluated. Cigarette smoke extract (CSE)-induced senescence model of airway epithelial cells was treated with ECC-BYF III, and related enzymes and proteins involved in oxidative stress and mitophagy were detected.

**Results:**

ECC-BYF III markedly rescued pulmonary function and histopathological changes, which might be associated with the amelioration of lung senescence, including the reduction of malondialdehyde (MDA) and tumor necrosis factor-α (TNF-α), interleukin (IL)-6 and matrix metalloproteinase (MMP)-9 levels, increase of the level in total superoxide dismutase (T-SOD), and decease in the p21 level in the airways. Furthermore, ECC-BYF III suppressed p16 and p21 expressions and senescence-associated β-galactosidase (SA-β-Gal) in CSE-induced airway epithelial cells. Moreover, ECC-BYF III upregulated mitophagy-related proteins, including the co-localizations of TOM20 and LC3B, PINK1 and PARK2, and improved mitochondrial function by upregulating mitochondrial mitofusin (MFN)2 and reducing dynamin-related protein 1 (DRP1) expression. ECC-BYF III enhanced the activities of T-SOD and GSH-PX by up-regulating NRF2, thus inhibiting oxidative stress. After intervention with NRF2 inhibitor, the regulation effects of ECC-BYF III on oxidative stress, mitophagy and senescence in airway epithelial cells were significantly suppressed.

**Conclusions:**

ECC-BYF III exerts beneficial effects on COPD rats by ameliorating airway epithelial cell senescence, which is mediated by inhibiting oxidative stress and subsequently enhancing mitophagy through the activation of NRF2 signaling.

**Supplementary Information:**

The online version contains supplementary material available at 10.1186/s12890-022-02191-9.

## Background

Chronic obstructive pulmonary disease (COPD) is a heterogeneous disorder that involves irreversible airflow restriction and chronic abnormal inflammatory reaction to harmful particles or gases [1, 2]. COPD is the third-leading cause of death worldwide, with a high incidence and disability rate, and it is accompanied by significant economic and social pressure [1, 3]. Traditional Chinese medicine (TCM) therapies have been widely used for stable COPD without or with mild side effects and are gaining increasing attention for their significant effects.

Bufei Yishen formula (BYF), a Chinese herbal formula, exhibited good efficacy on COPD clinical symptoms including the reduction of the frequency of acute exacerbation in our previous study [4]. However, the complex ingredients of BYF make for elucidating the mechanisms involved difficult and hinder international promotion. Therefore, five effective components were identified from herbal medicines of BYF based on in vivo experiments, combined in a fixed ratio as the effective-component compatibility of BYF III (ECC-BYF III), which suppressed inflammation by regulating p65, JNK, and p38 in COPD [5].

Cell senescence in COPD, particularly in alveolar and airway epithelial cells, increases the risk of respiratory tract infection, aggravates progressive emphysema, and may lead to airway remodeling, which can be induced by extracellular or intracellular stimuli such as telomere attrition (replicative senescence), irreparable DNA damage, mitochondrial dysfunction, and oxidative stress (excessive senescence) [2, 6–11]. Cell senescence is always accompanied by a complex phenotype, such as altered cell morphology, cell cycle arrest, increased senescence-associated β-galactosidase (SA-β-gal), and senescence-associated secretory phenotype (SASP), which is primarily mediated by the p53 or p16/p21 pathway [11].

Mitophagy is a type of elimination of irreversibly damaged mitochondria that helps slow cell senescence. It is regulated by phosphatase and tensin homolog-induced putative kinase 1 (PINK1) and Parkin (PARK) 2. Studies have revealed that oxidative stress may result in insufficient mitophagy by downregulating the expressions of PINK1 and PARK2 [8, 12–15], and elevated nuclear factor erythroid-2 related factor 2 (NRF2) can alter the process [16, 17].

Therefore, in this study, we explored the effects of ECC-BYF III on COPD rats, and investigated the mechanism by which ECC-BYF III improved cigarette smoke extract (CSE)-induced airway epithelial cell senescence by suppressing oxidative stress and subsequently enhancing insufficient mitophagy.

## Materials and methods

### Animals

Forty-eight Sprague-Dawley rats (24 male and 24 female, 200 ± 20 g) were purchased from the Laboratory Animal Center of Henan Province (Zhengzhou, China).

### Drugs

The components of ECC-BYF III were purchased from Manster Biotechnology co., LTD (Chengdu, China). N-acetylcysteine (Flumucil, as a positive control in the animal experiments) was purchased from Zambon Pharmaceutical Co., Ltd., (Hainan, China). Luteolin (purity: ≥ 98%, 491–70-3) was provided by Manster Biotechnology Co., Ltd.

### COPD model and administration

After being acclimatized for 7 days, rats were randomized into the normal, model, ECC-BYF III and NAC groups (half male and half female in each group). The COPD model was established with tobacco smoke exposure and bacterial infection from week 1 to week 8. Briefly, the model rats were exposed to tobacco smoke (smoke concentrations, 3000 ± 500 ppm, 1.0 mg of nicotine, 11 mg CO and 10 mg of tar per cigarette; Hongqiqu® filter cigarettes, Zhengzhou, China) for 40 min twice a day, and *Klebsiella pneumoniae* (0.1 mL, 6 × 10^8^ CFU/mL; bacterial strain: 46114; National Center for Medical Culture Collection, Beijing, China) once every 5 days [18].

From 9 to 16 weeks, the treatment groups (drug-treated COPD model rats) underwent oral gavage with ECC-BYF III (dosage: 6.48 mg/kg, q.d.) or NAC (dosage: 54 mg/kg, q.d.). Meanwhile, normal rats underwent oral gavage with saline once a day. The dosage of ECC-BYF III and NAC were decided and adjusted weekly based on the following formula: D_rat_ = D_human_ × (K_rat_ / K_human_) × (W_rat_ / W_human_)^2/3^ (D, dosage; K, body shape index; W, body weight). At week 16, the rats were sacrificed utilizing 2% sodium pentobarbital (40 mg/kg) and samples were obtained.

### Pulmonary function analysis

Pulmonary function was measured in unrestrained rats with whole body plethysmograph (WBP) system (Buxco Inc., Wilmington, NC, USA) every 4 weeks. The relevant continuous ventilatory parameters, including tidal volume (V_T_), minute volume (MV), peak expiratory flow (PEF), and expiratory flow at 50% tidal volume (EF50) were calculated. A FinePointeTM pulmonary function test system (Buxco Inc., USA) was used to measure the forced vital capacity (FVC), forced expiratory volume at 0.3 s (FEV 0.3), airway resistance (RI) and dynamic lung compliance (Cydn).

### Histopathology analysis

The left lobe was fixed in 10% paraformaldehyde solution for 72 h. Then, 3 mm of the lobe near- the lung hilum was cut for dehydration and fixed in paraffin to make wax blocks and subsequently cut into 3 μm - sections for hematoxylin–eosin (H&E) staining. Thereafter, the stained tissue sections were taken using optical microscopy and a photographic system (Olympus Optical Co., Ltd., Japan). Six images were selected for each group and six fields were selected for each image randomly. Moreover, the alveolar mean linear intercept (MLI, μm) and the mean alveolar number (MAN, /mm^2^) [19, 20] were counted using the counting tool of Adobe Photoshop CC software to evaluate the degree of emphysema.

### Kit analysis

The concentration of tumor necrosis factor-α (TNF-α), interleukin (IL)-6, and matrix metalloproteinase (MMP)-9 in the bronchoalveolar lavage fluid (BALF) were quantified by enzyme-linked immunosorbent assay kits (Boster Bio-Engineering Co., Ltd., Wuhan, China). Meanwhile, the total superoxide dismutase (T-SOD), malondialdehyde (MDA), and glutathione peroxidase (GSH-PX) levels in lung tissues or cells were measured with kits (Elabscience, Wuhan, China) according to the manufacturer^’^s protocol.

### Immunofluorescence analysis

The expression of p21 in lungs, especially in the bronchus, was detected by immunofluorescence. After dewaxing and dehydration treatment, the lung tissue slices were added with a 5% BSA for 30 min, and then anti-p21 antibody was incubated (1:1000 dilution; Cell Signaling Technology (CST), MA, USA) overnight at 4 °C. On the following day, slices were incubated with Cy3–conjugated Affinipure goat Anti-Rabbit IgG (H + L) (Proteintech, Wuhan, China) for 50 min. At least six images were randomly taken for each section and the red staining area was calculated by CaseViewer software.

Moreover, BEAS-2B cells cultured in 24-well culture slides were fixed with 4% paraformaldehyde for 15 min and blocked with sheep serum protein mixed with 0.3% TritonX-100 for 2 h at room temperature (26 °C). The primary antibody LC3B (GTX17380, Gene Tex), TOM20 (11802–1-AP, Proteintech) and the secondary antibody were incubated according to the manufacturer’s instruction. Finally, confocal laser scanning microscopy was used to image and assess the mitophagy.

### Preparation of cigarette extract

The mainstream cigarette smoke was sucked into a 50 mL syringe, and then slowly injected into the serum-free Dulbecco’s modified eagle medium (DMEM). The optical density at 320 nm wavelength was measured with a spectrophotometer, and adjusted to 1.8–2.0 with DMEM. Subsequently, the prepared CSE solution was filtered (0.22 μM) to obtain a 100% CSE solution.

### Cell culture and treatment

BEAS-2B (ATCC) cells, a human bronchial cell line, were cultured in DMEM (Solarbio, Science & Technology Co., Ltd., Beijing, China) with 10% fetal bovine serum (FBS). Briefly, after being maintained in FBS-free medium for 3 h, the cells were pre-incubated with NRF2 inhibitor for 2 h, and then incubated with ECC-BYF III at different concentrations (35, 17.5, 8.75 μg/mL) for 3 h, and 10%CSE was added subsequently. The cells were collected after 6 h.

### SA-β-gal staining

SA-β-Gal staining was conducted following the manufacturer’s instruction (G1580, Solarbio, Science & Technology Co., Ltd.). Briefly, BEAS-2B cells cultured in 6-well culture slides were fixed with 1 mL of β-Gal stationary liquids for 15 min at room temperature (26 °C), washed three times with phosphate-buffered saline (PBS), and then incubated with β-Gal staining at 37 °C overnight. The proportion of the stained cells (green staining cells) to total BEAS-2B cells was calculated.

### Reactive oxygen species analysis

BEAS-2B cells were suspended with PBS mixed with the DCFH-DA fluorescent probe (E-BC-K138-F, Elabscience) (10 μM) and incubated at 37 °C for 30 min. The cells were washed twice with PBS and then resuspended with 300 μL of PBS. Thereafter, the fluorescent intensity was measured by the FACS CantoTM II (BD Biosciences, San Jose, CA, USA).

### Electron microscopy

BEAS-2B cells were fixed with 2.5% glutaraldehyde (P1126S, Solarbio, Science and Technology Co., Ltd.) overnight at 4 °C. Sample formulation and electron microscope photography were completed by the electron microscope at the Center of Henan University of Traditional Chinese Medicine. Mitochondria and autophagosomes were assessed.

### Western blotting

The cells were lysed in a radioimmunoprecipitation assay lysis mixing protease inhibitor and phosphatase inhibitor. Protein concentration was determined using the BCA protein assay kit (PC0020, Solarbio, Science and Technology Co., Ltd.). The protein samples were separated in 10% sodium dodecyl sulfate-polyacrylamide gel electrophoresis and transferred to polyvinylidene difluoride membranes. The membranes were blocked with 5% skimmed milk in TBST at room temperature for 1 h and incubated with the primary antibodies overnight at 4 °C. The specific primary antibodies were β-actin (1:1000 diluted; Proteintech), p21 (1:1000 diluted; CST), p16(1:1000 diluted; Proteintech), PINK1 (1:1000 diluted; Proteintech), PARK2 (1:1000 diluted; Proteintech), MFN2 (1:1000 diluted; CST), DRP1 (1:1000 diluted; CST), NRF2 (1:1000 diluted; Gene Tex), HO-1 (1:1000 diluted; CST). Subsequently, the membranes were incubated with secondary antibodies of HRP-conjugated goat anti-mouse and anti-rabbit (1:5000 diluted, Proteintech) for 1 h at room temperature. After Washing three times with TBST, the bands were visualized with an enhanced chemiluminescence reagent. The interest protein band intensities were adjusted with β-actin control intensities. Thereafter, grayscale values were analyzed using Image Lab software.

### Statistical analysis

All data were processed by IBM SPSS Statistics for Windows, Version 22.0 (IBM Corp., Armonk, NY, USA) and graphed with GraphPad Prism 10.0. Data were presented as means ± standard deviation. Significant differences were assessed by one-way analysis of variance followed by Tukey’s test where appropriate. Values of *P* < 0.05 were considered significant.

## Results

### Effect of ECC-BYF III on pulmonary function and histopathological changes in COPD rats

**Fig. 1:**
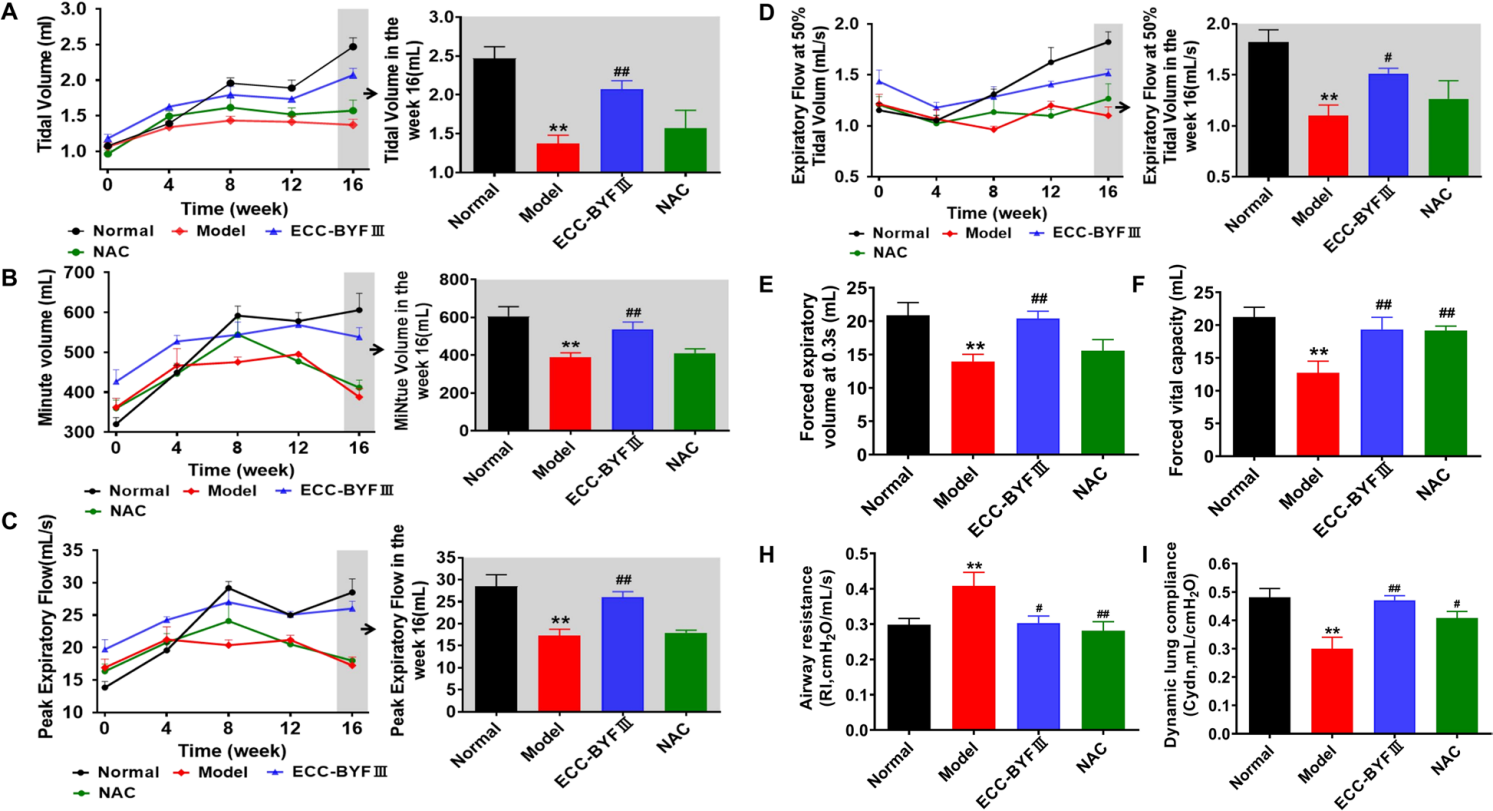
Effect of ECC-BYF III on pulmonary function of COPD rats. ECC-BYF III (dosage: 6.48 mg/kg, q.d) or N-Acetylcysteine (dosage: 54 mg/kg, q.d) was given treatment groups by intragastric administration from 9 to 16 weeks. **A** Changes of V_T_ in all groups. **B** Changes of MV in all groups. **C** Changes of PEF in all groups. **D** Changes of EF50 in all groups. **E** Changes of FEV0.3 in all groups. **F** Changes of FVC in all groups. **H** Changes of RI in all groups. **I** Changes of Cydn in all groups. All data are expressed as the mean ± SE, (*n* = 6). Animals are randomly selected. ^**^P < 0.01 vs. the normal group; ^#^*P* < 0.05 vs. the model group, ^##^*P* < 0.01 vs. the model group

**Fig. 2:**
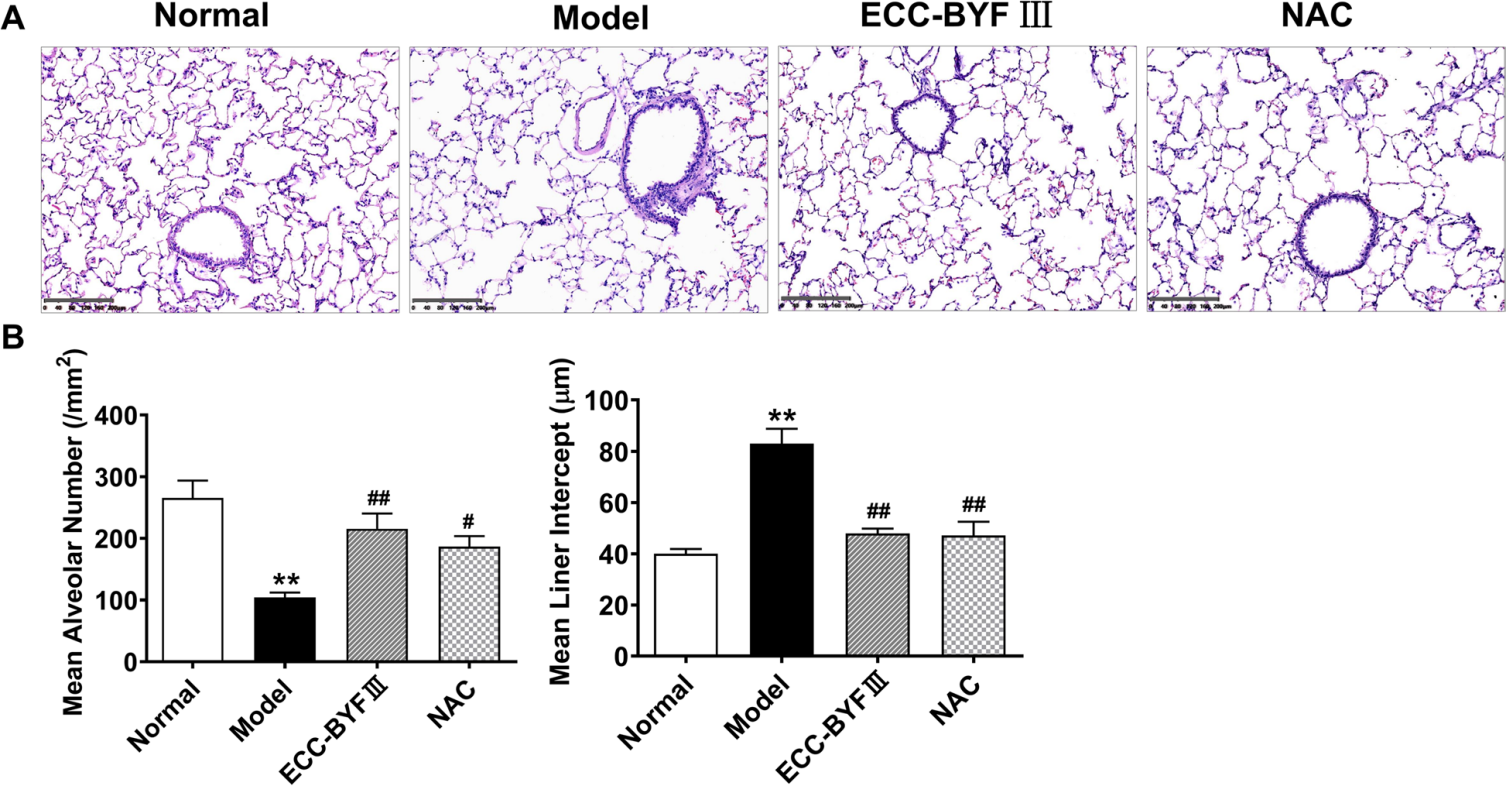
Effect of ECC-BYF III on histopathological changes of COPD rats. **A** H&E staining of lungs from the different treatment groups (× 100). Bar: 200 μm. **B** Changes of MAN in different groups. **C** Changes of MLI in the different groups. The data are expressed as the mean ± SE, (*n* = 6). Animals are randomly selected. ^**^P < 0.01 vs. the normal group; ^#^*P* < 0.05 vs. the model group, ^##^*P* < 0.01 vs. the model group

The severity of COPD was determined by pulmonary function and histopathological changes. VT, MV, PEF, and EF50 were all significantly lower in COPD rats, as were FVC, FEV 0.3 and Cydn, while the airway resistance was elevated, as presented in Fig. 1. Treatment with ECC-BYF III and NAC significantly improved lung function. Figure 2 depicts the histopathological changes in COPD, including alveolar and bronchial thickening, emphysema, and inflammatory cell infiltration. Quantitative analysis of MAN and MLI of section sampling revealed that an increase in MLI was accompanied by a decrease in MAN. Treatment with ECC-BYF III and NAC could effectively rescue the histopathological changes above. These data suggested that ECC-BYF III could attenuate COPD rats.

### Effect of ECC-BYF III on cell SASP in COPD rats

**Fig. 3:**
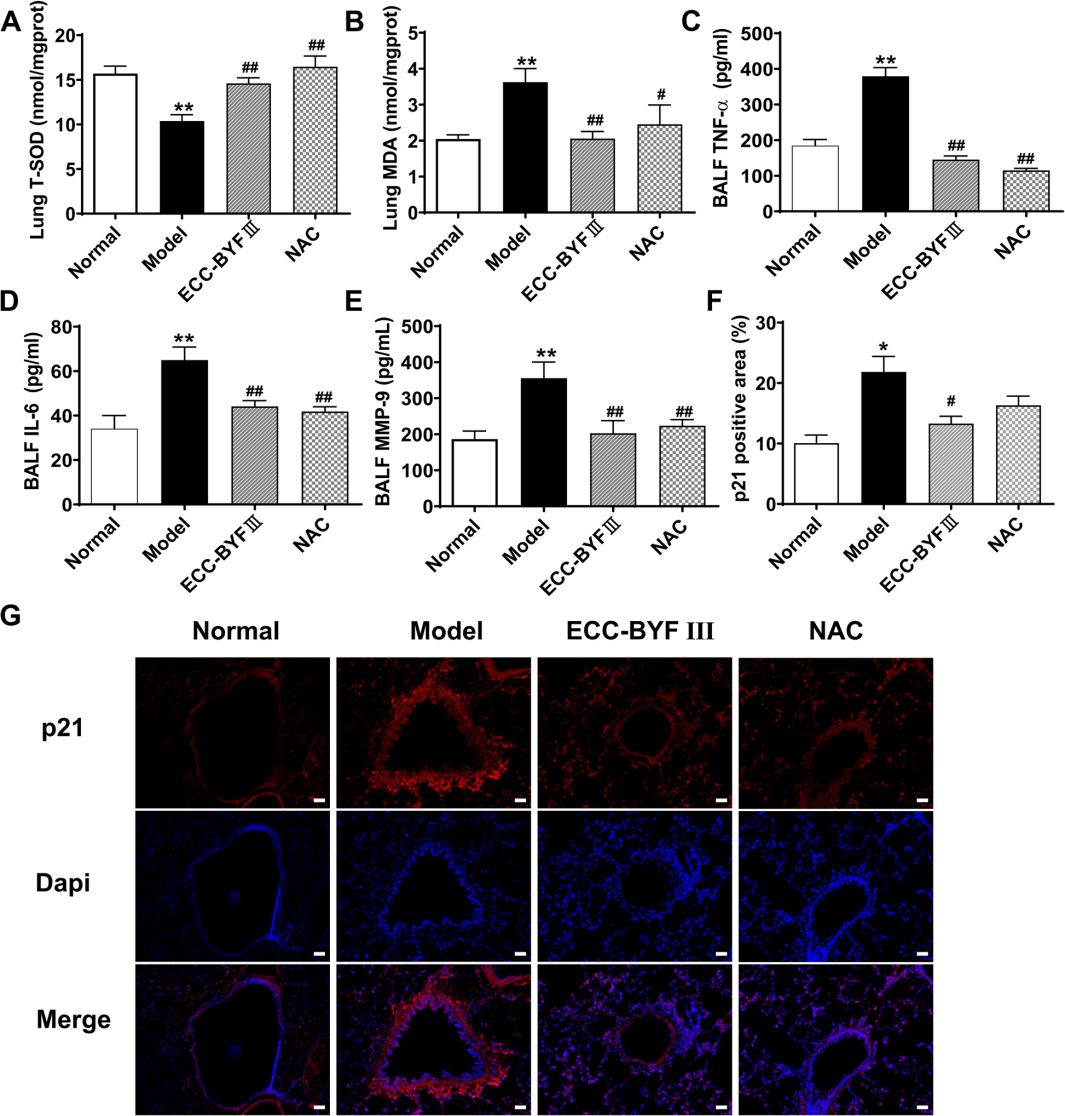
Effect of ECC-BYF III on cell senescence**-**associated secretory phenotype in COPD rats. **A, B** The expression of T-SOD and MDA in lung tissue; **C, D** The expression of inflammation cytokines in BALF; **E** The expression of MMP-9 in BALF; **F** The expression of p21 positive area (%). The data are expressed as the mean ± SE, (*n* = 6). Animals are randomly selected. ^**^P < 0.01 vs. the normal group, ^*^P < 0.05 vs. the normal group; ^##^*P* < 0.01 vs. the model group, ^#^*P* < 0.05 vs. the model group; **G** immunofluorescent staining of p21, and red-stained represents the expression of p21, (magnification, × 200). Bar: 50 μm

Lung cell senescence results in the release of SASP such as inflammatory cytokines (IL-1β, IL-6, and TNF-α), chemokines (CXCL-1, CXCL-8, and CCL-2), growth factors, and proteases (MMP-2 and MMP-9). It also induces cell cycle arrest, decreased antioxidant capacity (T-SOD lower), and damaged-associated molecular patterns [6, 11, 21, 22]. MDA activity, MMP-9 level, and TNF-α and IL-6 expressions were all suppressed after ECC-BYF III and NAC administration, whereas T-SOD expression was increased (Fig. 3A–E). According to the immunofluorescence results, ECC-BYF III treatment significantly suppressed the expression of p21, a cyclin-dependent kinase inhibitor, indicating cell senescence with persistently elevated expression (Fig. 3F and G). These findings suggested that ECC-BYF III treatment improved several SASPs in the lungs of COPD rats.

### Effect of ECC-BYF III on cell senescence in BEAS-2B cells induced by CSE

**Fig. 4:**
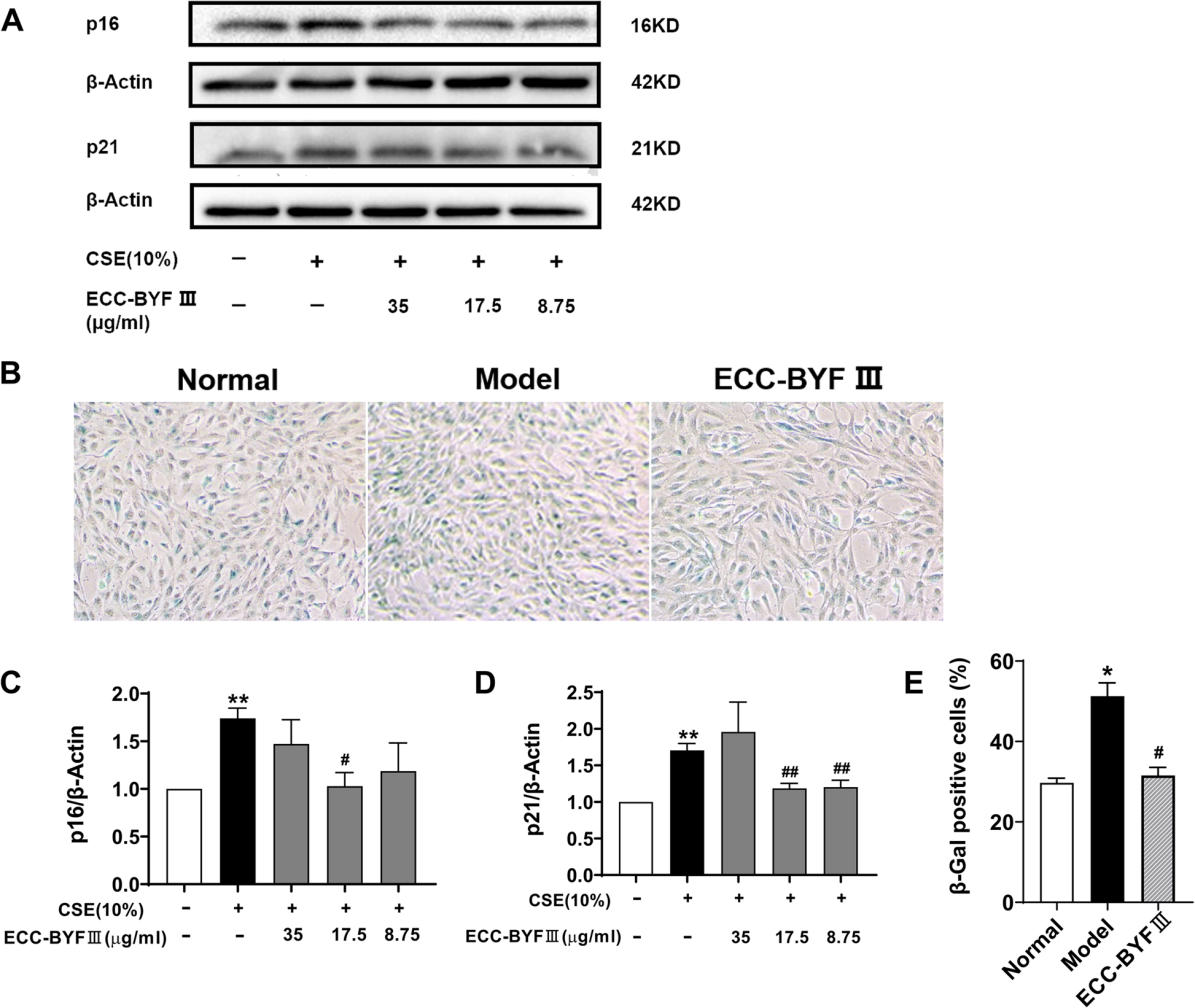
Effects of ECC-BYF III on cells senescence in CSE-induced BEAS-2B. **A** The total protein expressions of p21 and p16; Full-length gels are presented in Supplementary Fig. 1 (**B**) SA-β-Gal staining of different groups, the dosage of ECC-BYF III (17.5 μg/ml) was used. The level of (**C**) p16 (**D**) p21 and (**E**) a histogram of percentage of positive cells. The data are expressed as the mean ± SE, (*n* = 3–6). ^**^P < 0.01 vs. the normal group, ^*^P < 0.05 vs. the normal group; ^##^*P* < 0.01 vs. the model group, ^#^*P* < 0.05 vs. the model group

A previous study has found a high level of p21 expression in lung tissues, particularly in the airways of COPD rats. Cigarette smoke exposure is a major factor that causes elevated p21 expression, airway epithelial cell senescence, and COPD airway epithelial dysfunction. The expressions of p21, p16, and SA-β-Gal were detected in CSE-induced BEAS-2B cells to determine the effect of ECC-BYF III on cell senescence. We found that 10% CSE exposure increased the expressions of p21, p16, and SA-β-Gal. ECC-BYF III treatment led to a significant decrease in the expressions of p21 and p16 and the number of SA-β-Gal-stained cells (Fig. 4). These findings suggested that ECC-BYF III effectively suppressed CSE-induced airway epithelial cell senescence.

### ECC-BYF III upregulated mitophagy in BEAS-2B cells induced by CSE

**Fig. 5:**
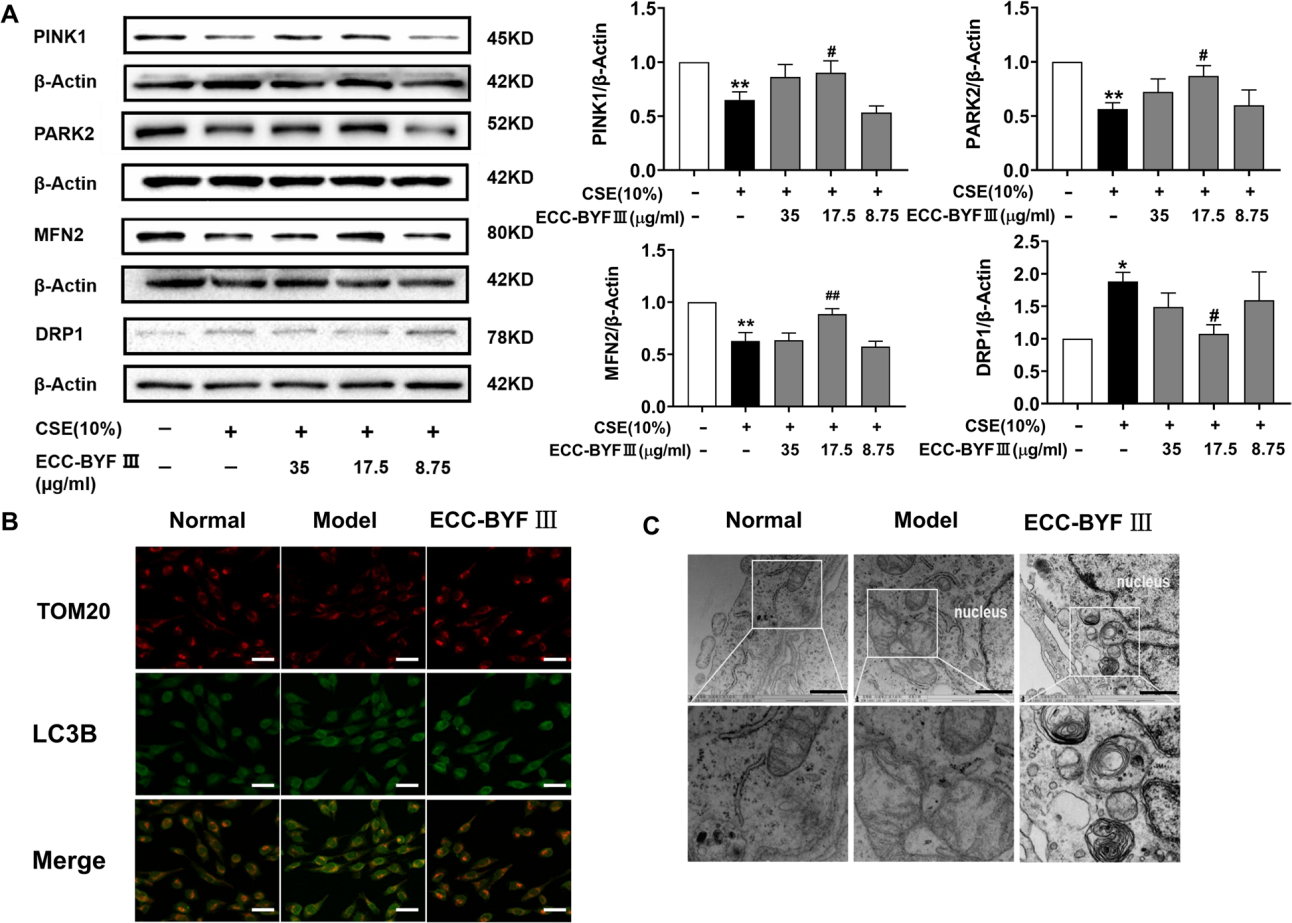
ECC-BYF III regulated CSE-induced mitophagy deficiency. **A** PINK1, PARK2, MFN2 and DRP1 levels as assessed by western blot; Full-length gels are presented in Supplementary Fig. 2; The data are expressed as the mean ± SE, (*n* = 4). ^**^P < 0.01 vs. the normal group, ^*^P < 0.05 vs. the normal group; ^##^*P* < 0.01 vs. the model group, ^#^*P* < 0.05 vs. the model group; **B** Immunofluorescent staining of the co-localization of TOM20 (yellow dots) and LC3B (green dots), (magnification, × 200); Bar: 50 μm. **C** Electron microscopy detection of mitochondria and mitophagy in BEAS-2Bs (magnification, × 30,000). Bar: 1 μm

Mitophagy deficiency is a critical inducer of the accumulation of damaged mitochondrial and subsequent cell senescence. Thus, we assessed mitophagy in BEAS-2B cells induced by CSE or ECC-BYF III. We found that ECC-BYF III could significantly increase the expressions of PINK1 and PARK2 and intensify the co-localization of TOM20-labeled mitochondria, as indicated by the yellow dots and green dots (LC3B) in Fig. 5B. Furthermore, ECC-BYF III treatment improved mitochondrial swelling, decreased the number of damaged mitochondria, and increased the number of autophagosomes and lysosomes. Meanwhile, the expression of MFN2 increased and that of DRP1 decreased. Taken together, ECC-BYF III exhibited an effective role in enhancing mitophagy, subsequently eliminating damaged mitochondria, and improving mitochondrial function (Fig. 5).

### ECC-BYF III improved CSE-induced oxidative stress in BEAS-2B cells

**Fig. 6:**
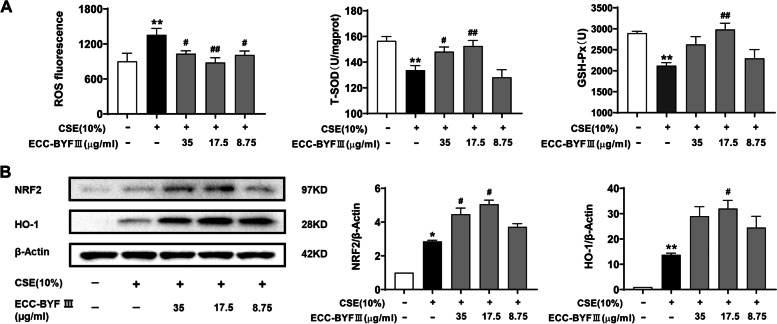
Effects of ECC-BYF III on mitophagy and oxidative stress. **A** ROS levels and the activity of T-SOD and GSH-PX; **B** The protein expression of NRF2 and HO-1; Full-length gels are presented in Supplementary Fig. 3. The data are expressed as the mean ± SE, (*n* = 3–7). ^**^P < 0.01 vs. the normal group, ^*^P < 0.05 vs. the normal group; ^##^*P* < 0.01 vs. the model group, ^#^*P* < 0.05 vs. the model group

CSE-induced insufficient mitophagy and abundant damaged mitochondria are frequently caused by oxidative stress [23]. After CSE exposure, ROS increased, whereas the activities of T-SOD and GSH-PX were downregulated in BEAS-2B cells. ROS was reduced by ECC-BYF III treatment, whereas the activities of T-SOD and GSH-PX were increased in BEAS-2B cells (Fig. 6A). Furthermore, we discovered that ECC-BYF III treatment significantly increased NRF2 and HO-1 (Fig. 6B). The findings suggested that ECC-BYF III could activate NRF2 and prevent CSE-induced oxidative stress.

### Involvement of NRF2 signal in ECC-BYF III’s anti- senescence effect

**Fig. 7:**
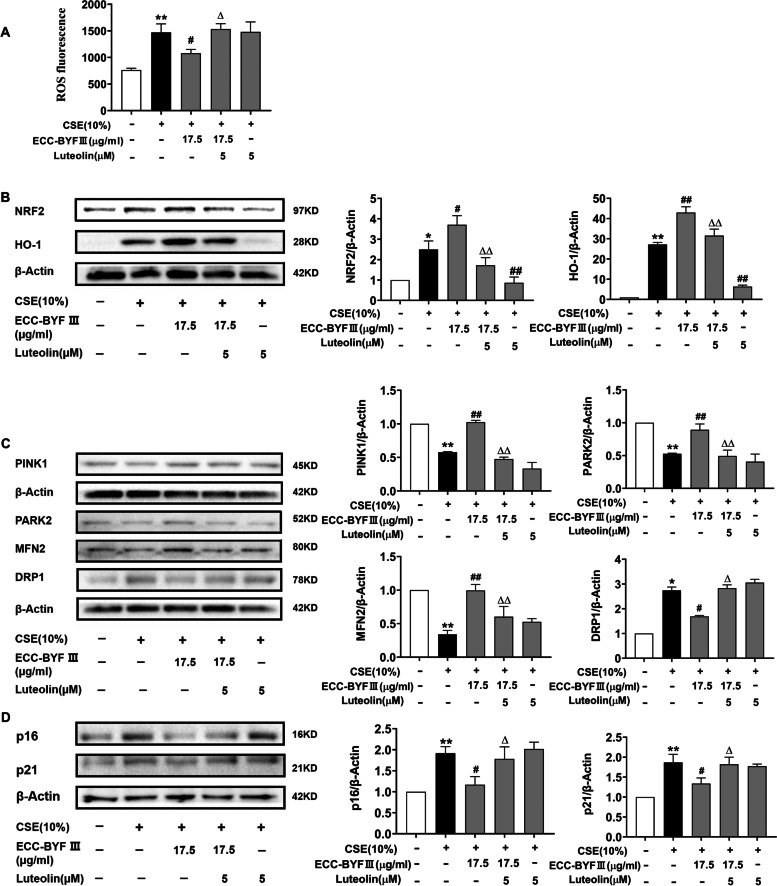
ECC-BYF III improve CSE-induced excessive cell senescence by suppressing oxidative stress, strengthening mitophagy. **A** The fluorescence intensity of ROS after intervention with Luteolin; **B** The protein expressions of NRF2 and HO-1 after intervention with Luteolin. Full-length gels are presented in Supplementary Fig. 4. **C** The protein expressions of PINK1, PARK2, MFN2, DRP1 after intervention with Luteolin; Full-length gels are presented in Supplementary Fig. 5. **D** The protein expressions of p16 and p21 after intervention with Luteolin. Full-length gels are presented in Supplementary Fig. 6. The data are expressed as the mean ± SE, (*n* = 3). ^**^P < 0.01 vs. the normal group, ^*^P < 0.05 vs. the normal group; ^##^*P* < 0.01 vs. the model group, ^#^*P* < 0.05 vs. the model group; ^△△^*P* < 0.01 vs. the ECC-BYF III treatment group, ^△^*P* < 0.05 vs. the ECC-BYF III treatment group

To investigate the role of NRF2 in the anti-senescence effect of ECC-BYF III, we treated BEAS-2B cells with NRF2 inhibitors and/or ECC-BYF III. We discovered that co-treatment with luteolin (NRF2 inhibitor) inhibited the effects of ECC-BYF III, which could be reflected by the downregulation of NRF2 and HO-1 and higher ROS activity, as well as by the inhibition the activation of related proteins (PINK1, PARK2 and MFN2) and significant upregulation of the expression of DRP1. Luteolin eventually inhibited ECC-BYF III’s ability to reduce the expressions of p16 and p21 (Fig. 7). All data demonstrated that ECC-BYF III may play a role in ameliorating cell senescence by inhibiting oxidative stress and mitochondrial function through NRF2 signaling.

## Discussion

COPD is a major social problem that endangers public health because of its high morbidity and mortality rates. TCM is widely acknowledged for its efficacy in the treatment of COPD. The original BYF, which included 12 Chinese medicines, had a positive effect on COPD clinical symptoms [4, 24]. However, the complexity of the ingredients makes elucidating the mechanisms involved difficult. The effective compounds were identified from BYF and combined with a fixed ratio to produce ECC-BYF III. 20-S-ginsenoside Rh1, astragaloside IV, icariin, nobiletin, and paeonol are among the five compounds found in ECC-BYF III [5, 25]. ECC-BYF III effectively rescued pulmonary function, lung histopathological changes, and SASP in COPD rats in this study. Furthermore, by inhibiting oxidative stress and promoting insufficient mitophagy, ECC-BYF III may alleviate CSE-induced airway epithelial cell senescence.

Lung senescence, mainly including alveolar and airway epithelial cell senescence, participates in the pathogenesis of COPD [2, 6]. In COPD, cigarette smoke is a major driver of cell senescence. The accumulation of senescent cells in the lung contributes to SASP acquisition, and senescence increases the susceptibility to infection, airway remodeling, and exacerbation of emphysema in COPD [8, 10, 26]. The SASP takes the form of cell cycle arrest, release of inflammatory cytokines, and proteases, and reduction of antioxidant capacity [27, 28]. Indeed, our studies found that SASP occurred in COPD rats, such as increased p21, TNF-α, IL-6, MMP-9, and MDA activitives and T-SOD level decreased significantly in the lung or BALF. ECC-BYF III suppressed pulmonary function reduction and histopathological changes, while improved SASP in COPD rats. Thus, ECC-BYF III exerted a distinct effect on COPD rats by ameliorating the airway epithelial cell senescence and SASP.

The first barrier against external stimuli are airway epithelial cells. CS typically promotes airway epithelial cell senescence, with elevated p16, p21, and SA-β-Gal expressions [29, 30]. Our findings revealed that ECC-BYF III can prevent CSE-induced airway epithelial cell senescence. Excessive oxidants/ROS produced by CS exposure can damage biological macromolecules and cause mitochondrial dysfunction [31, 32]. Given that mitochondria are the pivotal hub of energy production and the producer of ROS, substantial studies have proved the importance of mitochondrial dysfunction in CS-induced cell senescence [12, 33]. Mitophagy is a special form of autophagy where damaged mitochondria are eliminated to maintain mitochondrial function. Insufficient mitophagy leads to the accumulation of damaged mitochondria, and thus causes the deficiency of energy, which impels cell senescence [30, 34, 35]. evidence suggests that PINK1 and PARK2 recruitment is necessary for mitophagy to ameliorate CSE-induced cell senescence, PINK1 knockdown noticeably reduced the expression of PARK2, and PARK2 knockdown could elevate the expressions of p21 and SA-β-Gal in response to CSE exposure [12, 15, 30].

In this study, we found that ECC-BYF III could inhibit ROS production induced by CSE, and enhance mitophagy by increasing the levels of PINK1 and PARK2. Bharathi et al. pointed out that CSE-induced mitochondrial fragmentation and dysfunction as marked by decreasing MFN2 and increasing DRP1 [36]. Treatment with ECC-BYF III could increase the expression of MFN2 and reduce DRP1, and alleviate the swelling and cristae disruption of mitochondria in CSE-induced airway epithelial cells with the increase in autophagosomes and lysosomes. This is the result of ECC-BYF III enhancing mitophagy and improving mitochondrial function. Thus, we hypothesized that ECC-BYF III reduced cell senescence by increasing the activities of PINK1 and PARK2. Since NRF2 is a common antioxidant factor and activation of NRF2 can protect cells from oxidative stress-induced mitophagy deficiency [16, 37, 38], we administered cells with NRF2 inhibitor and discovered that NRF2 inhibitor suppressed the inhibition effect of ECC-BYF III on ROS production, mitophagy deficiency, and cell senescence. Hence, targeting the NRF2 pathway to inhibit oxidative stress and intensify mitophagy is a potential mechanism for ECC-BYF III to ameliorate cell senescence.

## Conclusion

ECC-BYF III was effective in the treatment of COPD rats by ameliorating airway epithelial cell senescence. The effect was due to the inhibition of oxidative stress and the enhancement of mitophagy by NRF2/PINK1.

## Supplementary Information

**Additional file 1.**

**Additional file 2.**

**Additional file 3.**

**Additional file 4.**

**Additional file 5.**

**Additional file 6.**

**Additional file 7.**

## Data Availability

The datasets generated during and analyzed during the current study are not publicly available due to other study involving this data are in the progress, but are available from the corresponding author on reasonable request.

## Abbreviations

COPD: Chronic obstructive pulmonary disease
CSE: Cigarette smoke extract
Cydn: Dynamic lung compliance
DRP1: Dynamin-related protein 1
ECC-BYF III: Effective-component compatibility of Bufei Yishen formula III
EF50: Expiratory flow at 50% tidal volume
FEV0.3: Forced expiratory volume at 0.3 s
FVC: Forced vital capacity
GSH-PX: Glutathione peroxidase
IL-6: Interleukin 6
MAN: Mean alveolar number
MDA: Malondialdehyde
MFN: Mitofusin
MLI: Mean linear intercept
MMP-9: Matrix metalloproteinase
MV: Minute volume
NAC: N-Acetylcysteine
NRF2: Nuclear factor erythroid-2 related factor 2
PARK: Parkin
PEF: Peak expiratory
PINK1: PTEN-induced putative kinase 1
RI: Airway resistance
ROS: Reactive oxygen species
SASP: Senescence-associated secretory phenotype
SA-β-Gal: Senescence-associated β-galactosidase
TNF-α: Tumor necrosis factor-α
T-SOD: Total superoxide dismutase
V_T_: Tidal volume

## References

[1] 2021 GOLD Reports - Global Initiative for Chronic Obstructive Lung Disease - GOLD. Available from: https://goldcopd.org/2021-gold-reports/.

[2] Agusti A, Hogg JC (2019). Update on the pathogenesis of chronic obstructive pulmonary disease. N Engl J Med.

[3] Lopez-Campos JL, Tan W, Soriano JB (2016). Global burden of COPD. Respirology.

[4] Li JS, Li SY, Xie Y, Yu XQ, Wang MH, Sun ZK, Ma LJ, Jia XH, Zhang HL, Xu JP (2013). The effective evaluation on symptoms and quality of life of chronic obstructive pulmonary disease patients treated by comprehensive therapy based on traditional Chinese medicine patterns. Complement Ther Med.

[5] Li J, Xie Y, Zhao P, Qin Y, Oliver BG, Tian Y, Li S, Wang M, Liu X (2021). A chinese herbal formula ameliorates COPD by inhibiting the inflammatory response via downregulation of p65, JNK, and p38. Phytomedicine.

[6] Barnes PJ, Baker J, Donnelly LE (2019). Cellular senescence as a mechanism and target in chronic lung diseases. Am J Respir Crit Care Med.

[7] Munoz-Espin D, Serrano M (2014). Cellular senescence: from physiology to pathology. Nat Rev Mol Cell Biol.

[8] Vij N, Chandramani-Shivalingappa P, Van Westphal C, Hole R, Bodas M (2018). Cigarette smoke-induced autophagy impairment accelerates lung aging, COPD-emphysema exacerbations and pathogenesis. Am J Physiol Cell Physiol.

[9] Birch J, Anderson RK, Correia-Melo C, Jurk D, Hewitt G, Marques FM, Green NJ, Moisey E, Birrell MA, Belvisi MG (2015). DNA damage response at telomeres contributes to lung aging and chronic obstructive pulmonary disease. Am J Phys Lung Cell Mol Phys.

[10] Naikawadi RP, Disayabutr S, Mallavia B, Donne ML, Green G, La JL, Rock JR, Looney MR, Wolters PJ (2016). Telomere dysfunction in alveolar epithelial cells causes lung remodeling and fibrosis. JCI Insight.

[11] Coppe JP, Desprez PY, Krtolica A, Campisi J (2010). The senescence-associated secretory phenotype: the dark side of tumor suppression. Annu Rev Pathol.

[12] Yoo SM, Jung YK (2018). A molecular approach to Mitophagy and mitochondrial dynamics. Mol Cell.

[13] Sundar IK, Maremanda KP, Rahman I (2019). Mitochondrial dysfunction is associated with Miro1 reduction in lung epithelial cells by cigarette smoke. Toxicol Lett.

[14] Chen F, Liu Y, Wong NK, Xiao J, So KF (2017). Oxidative stress in stem cell aging. Cell Transplant.

[15] Sarraf SA, Sideris DP, Giagtzoglou N, Ni L, Kankel MW, Sen A, Bochicchio LE, Huang CH, Nussenzweig SC, Worley SH (2019). PINK1/Parkin influences cell cycle by sequestering TBK1 at damaged mitochondria, Inhibiting Mitosis. Cell Rep.

[16] Murata H, Takamatsu H, Liu S, Kataoka K, Huh NH, Sakaguchi M (2015). NRF2 regulates PINK1 expression under oxidative stress conditions. PLoS One.

[17] Silva-Palacios A, Ostolga-Chavarria M, Zazueta C, Konigsberg M (2018). Nrf2: molecular and epigenetic regulation during aging. Ageing Res Rev.

[18] Li Y, Li SY, Li JS, Deng L, Tian YG, Jiang SL, Wang Y, Wang YY (2012). A rat model for stable chronic obstructive pulmonary disease induced by cigarette smoke inhalation and repetitive bacterial infection. Biol Pharm Bull.

[19] Zhao YL, Li F, Liu YW, Shi YJ, Li ZH, Cao GK, Zhu W (2017). Adiponectin attenuates endoplasmic reticulum stress and alveolar epithelial apoptosis in COPD rats. Eur Rev Med Pharmacol Sci.

[20] Wang Y, Jiang X, Zhang L, Wang L, Li Z, Sun W (2014). Simvastatin mitigates functional and structural impairment of lung and right ventricle in a rat model of cigarette smoke-induced COPD. Int J Clin Exp Pathol.

[21] Tai H, Wang Z, Gong H, Han X, Zhou J, Wang X, Wei X, Ding Y, Huang N, Qin J (2017). Autophagy impairment with lysosomal and mitochondrial dysfunction is an important characteristic of oxidative stress-induced senescence. Autophagy.

[22] Dodig S, Cepelak I, Pavic I (2019). Hallmarks of senescence and aging. Biochem Med (Zagreb).

[23] Zhang Y, Xi X, Mei Y, Zhao X, Zhou L, Ma M, Liu S, Zha X, Yang Y (2019). High-glucose induces retinal pigment epithelium mitochondrial pathways of apoptosis and inhibits mitophagy by regulating ROS/PINK1/Parkin signal pathway. Biomed Pharmacother.

[24] Li SY, Li JS, Wang MH, Xie Y, Yu XQ, Sun ZK, Ma LJ, Zhang W, Zhang HL, Cao F (2012). Effects of comprehensive therapy based on traditional Chinese medicine patterns in stable chronic obstructive pulmonary disease: a four-center, open-label, randomized, controlled study. BMC Complement Altern Med.

[25] Li J, Ma J, Tian Y, Zhao P, Liu X, Dong H, Zheng W, Feng S, Zhang L, Wu M (2020). Effective-component compatibility of Bufei Yishen formula II inhibits mucus hypersecretion of chronic obstructive pulmonary disease rats by regulating EGFR/PI3K/mTOR signaling. J Ethnopharmacol.

[26] Adnot S, Amsellem V, Boyer L, Marcos E, Saker M, Houssaini A, Kebe K, Dagouassat M, Lipskaia L, Boczkowski J (2015). Telomere dysfunction and cell senescence in chronic lung diseases: therapeutic potential. Pharmacol Ther.

[27] Houssaini A, Breau M, Kebe K, Abid S, Marcos E, Lipskaia L, Rideau D, Parpaleix A, Huang J, Amsellem V (2018). mTOR pathway activation drives lung cell senescence and emphysema. JCI Insight.

[28] Baker JR, Donnelly LE, Barnes PJ (2020). Senotherapy: a new horizon for COPD therapy. Chest.

[29] Wu N, Yang D, Wu Z, Yan M, Zhang P, Liu Y (2019). Insulin in high concentration recede cigarette smoke extract induced cellular senescence of airway epithelial cell through autophagy pathway. Biochem Biophys Res Commun.

[30] Ito S, Araya J, Kurita Y, Kobayashi K, Takasaka N, Yoshida M, Hara H, Minagawa S, Wakui H, Fujii S (2015). PARK2-mediated mitophagy is involved in regulation of HBEC senescence in COPD pathogenesis. Autophagy.

[31] Fischer BM, Voynow JA, Ghio AJ (2015). COPD: balancing oxidants and antioxidants. Int J Chron Obstruct Pulmon Dis.

[32] Chen D, Kerr C (2019). The epigenetics of stem cell aging comes of age. Trends Cell Biol.

[33] Korolchuk VI, Miwa S, Carroll B, von Zglinicki T (2017). Mitochondria in cell senescence: is Mitophagy the weakest link?. EBioMedicine.

[34] Sachdeva K, Do DC, Zhang Y, Hu X, Chen J, Gao P (2019). Environmental exposures and asthma development: autophagy, Mitophagy, and cellular senescence. Front Immunol.

[35] Fujii S, Hara H, Araya J, Takasaka N, Kojima J, Ito S, Minagawa S, Yumino Y, Ishikawa T, Numata T (2012). Insufficient autophagy promotes bronchial epithelial cell senescence in chronic obstructive pulmonary disease. Oncoimmunology.

[36] Aravamudan B, Kiel A, Freeman M, Delmotte P, Thompson M, Vassallo R, Sieck GC, Pabelick CM, Prakash YS (2014). Cigarette smoke-induced mitochondrial fragmentation and dysfunction in human airway smooth muscle. Am J Phys Lung Cell Mol Phys.

[37] Dinkova-Kostova AT, Abramov AY (2015). The emerging role of Nrf2 in mitochondrial function. Free Radic Biol Med.

[38] Xiao L, Xu X, Zhang F, Wang M, Xu Y, Tang D, Wang J, Qin Y, Liu Y, Tang C (2017). The mitochondria-targeted antioxidant MitoQ ameliorated tubular injury mediated by mitophagy in diabetic kidney disease via Nrf2/PINK1. Redox Biol.

