# Supplementary figures and images for: Effective-component compatibility of Bufei Yishen formula III ameliorated COPD by improving airway epithelial cell senescence by promoting mitophagy via the NRF2/PINK1 pathway
Source: BMC Pulm Med. 2022 Nov 22;22:434. doi: 10.1186/s12890-022-02191-9 (PMC9682796; doi:10.1186/s12890-022-02191-9)

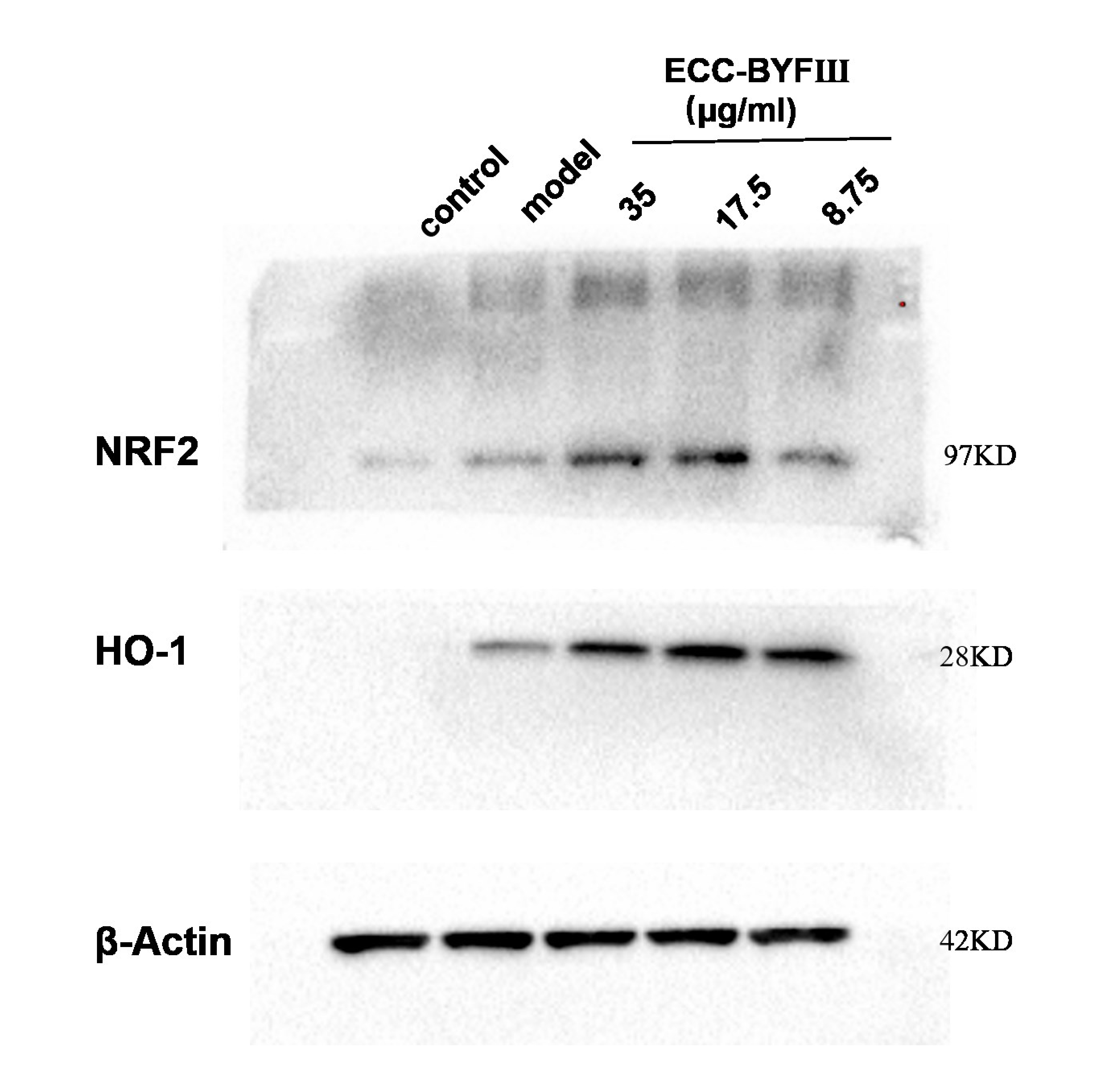

Supplement: Supplementary file 3 — Additional file 3. [file 12890_2022_2191_MOESM3_ESM.png]

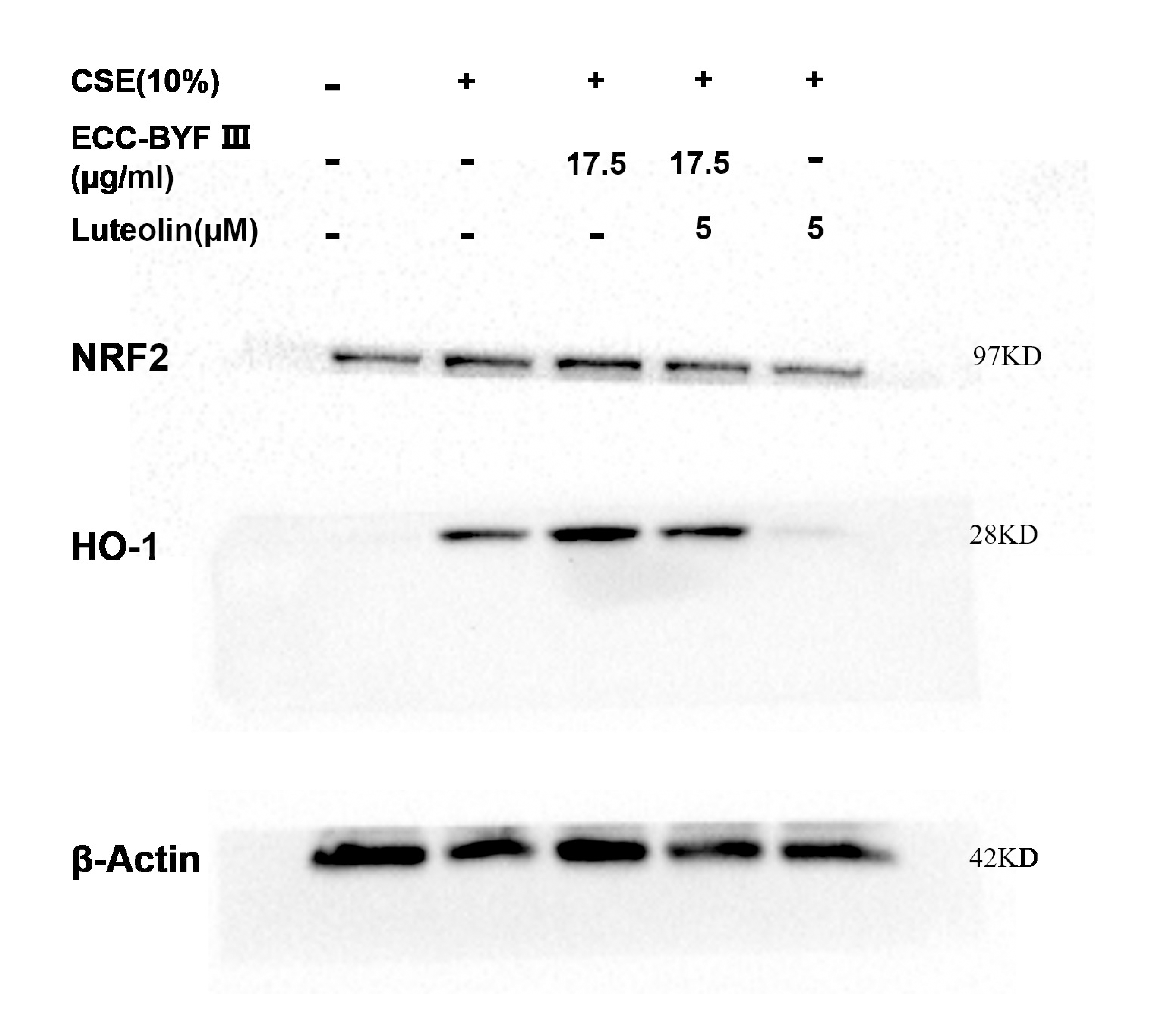

Supplement: Supplementary file 4 — Additional file 4. [file 12890_2022_2191_MOESM4_ESM.png]

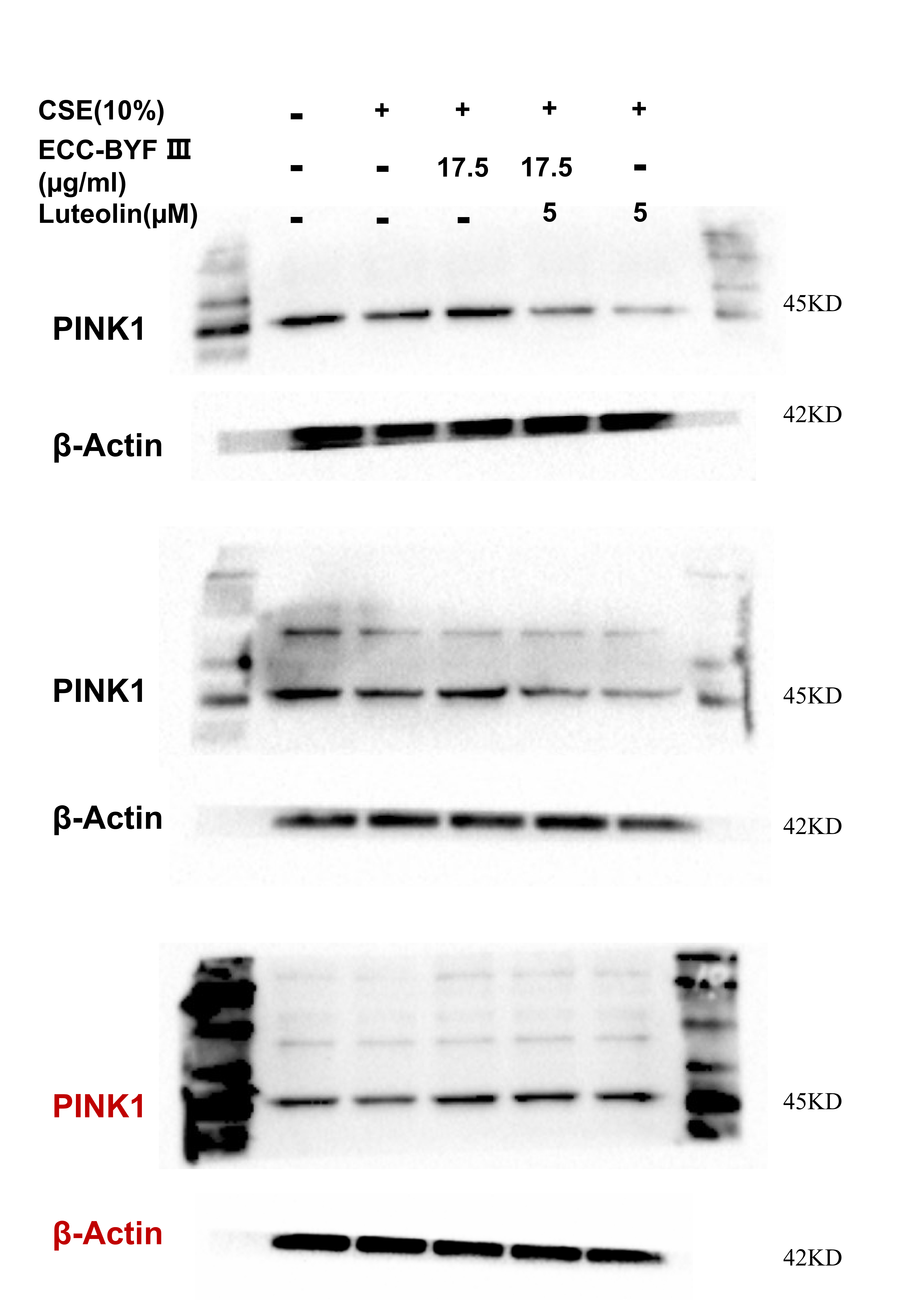

Supplement: Supplementary file 7 — Additional file 7. [file 12890_2022_2191_MOESM7_ESM.zip › Digital imange figure 11.png]

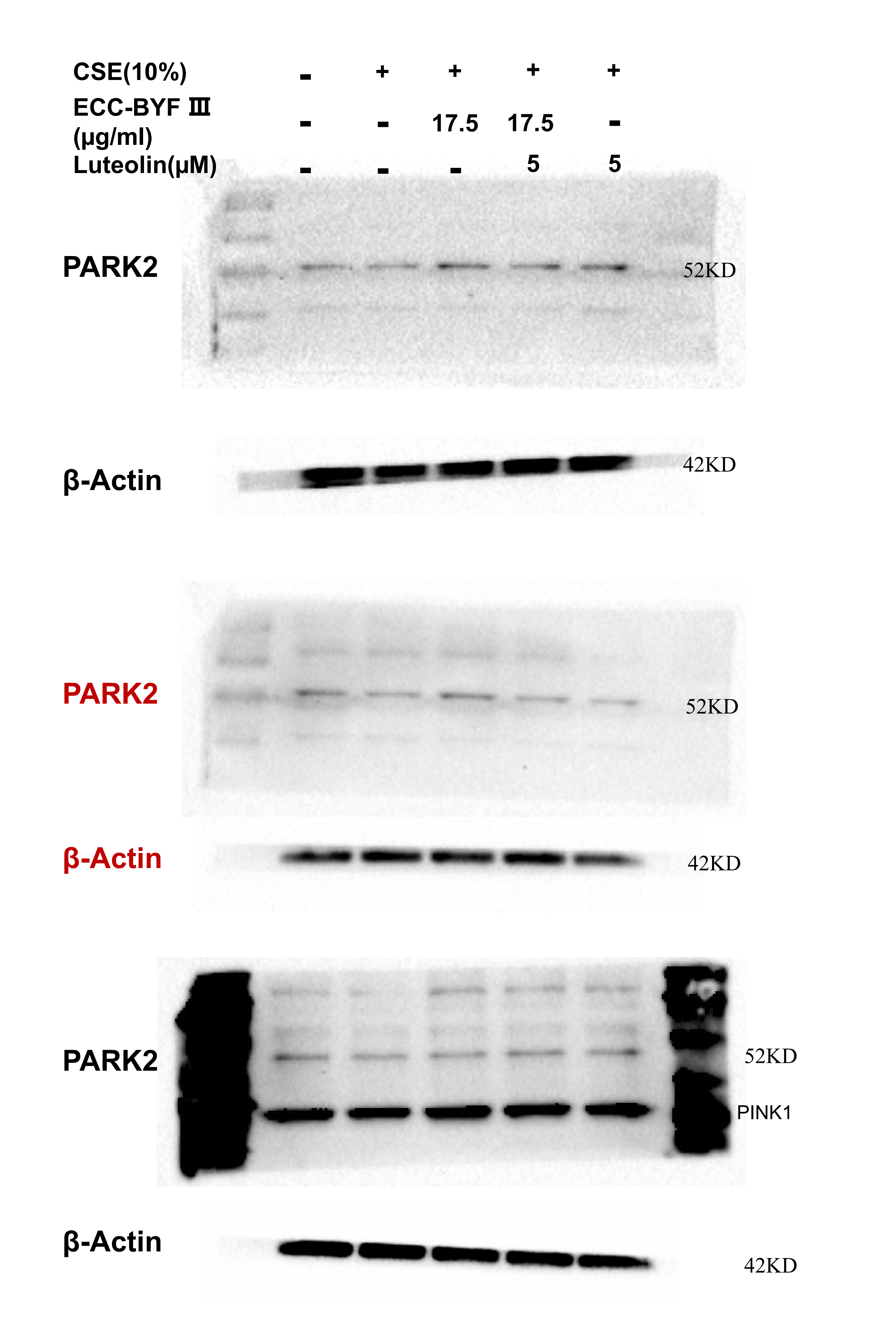

Supplement: Supplementary file 7 — Additional file 7. [file 12890_2022_2191_MOESM7_ESM.zip › Digital imange figure 12.png]

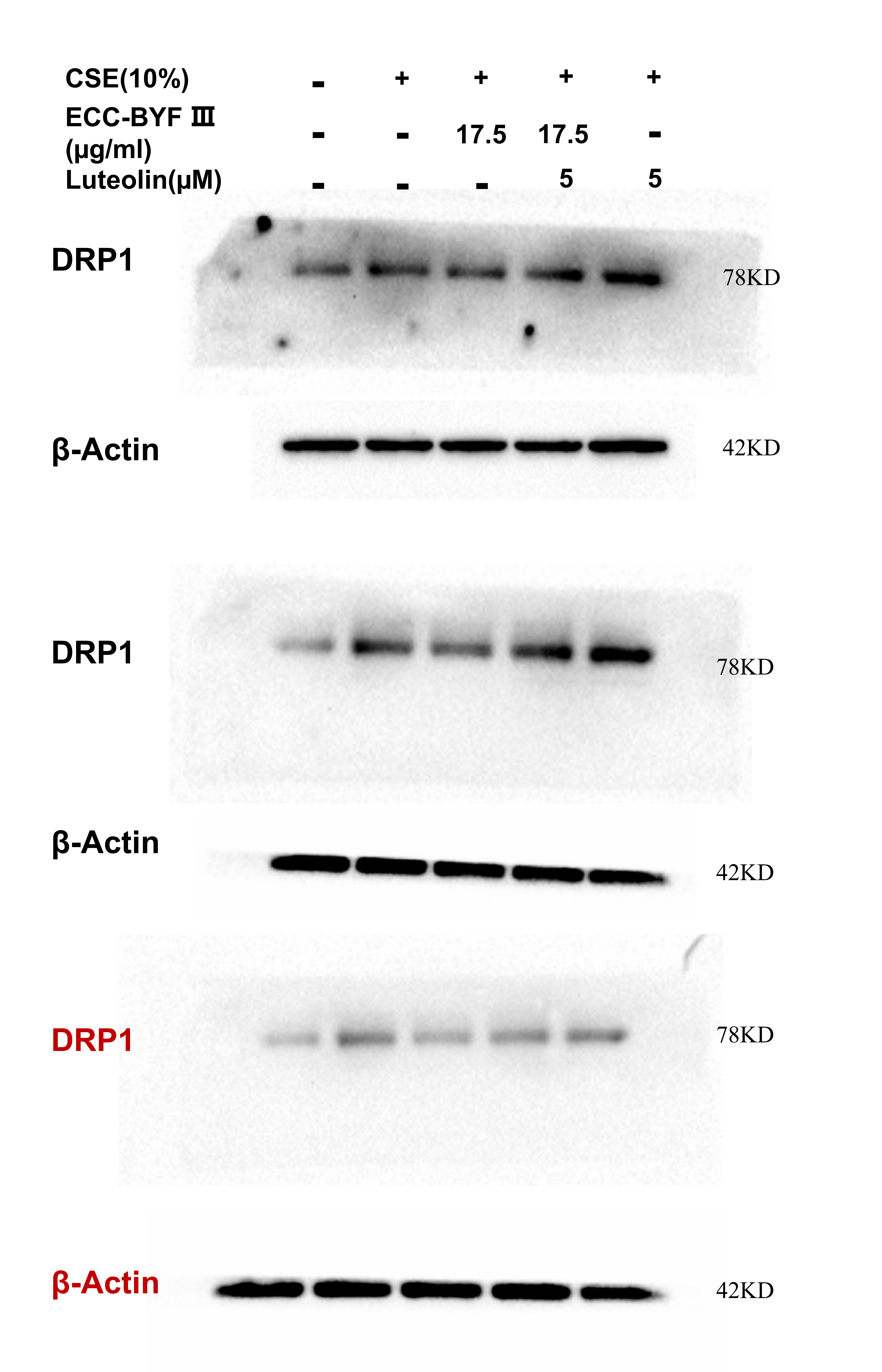

Supplement: Supplementary file 7 — Additional file 7. [file 12890_2022_2191_MOESM7_ESM.zip › Digital imange figure 14.png]

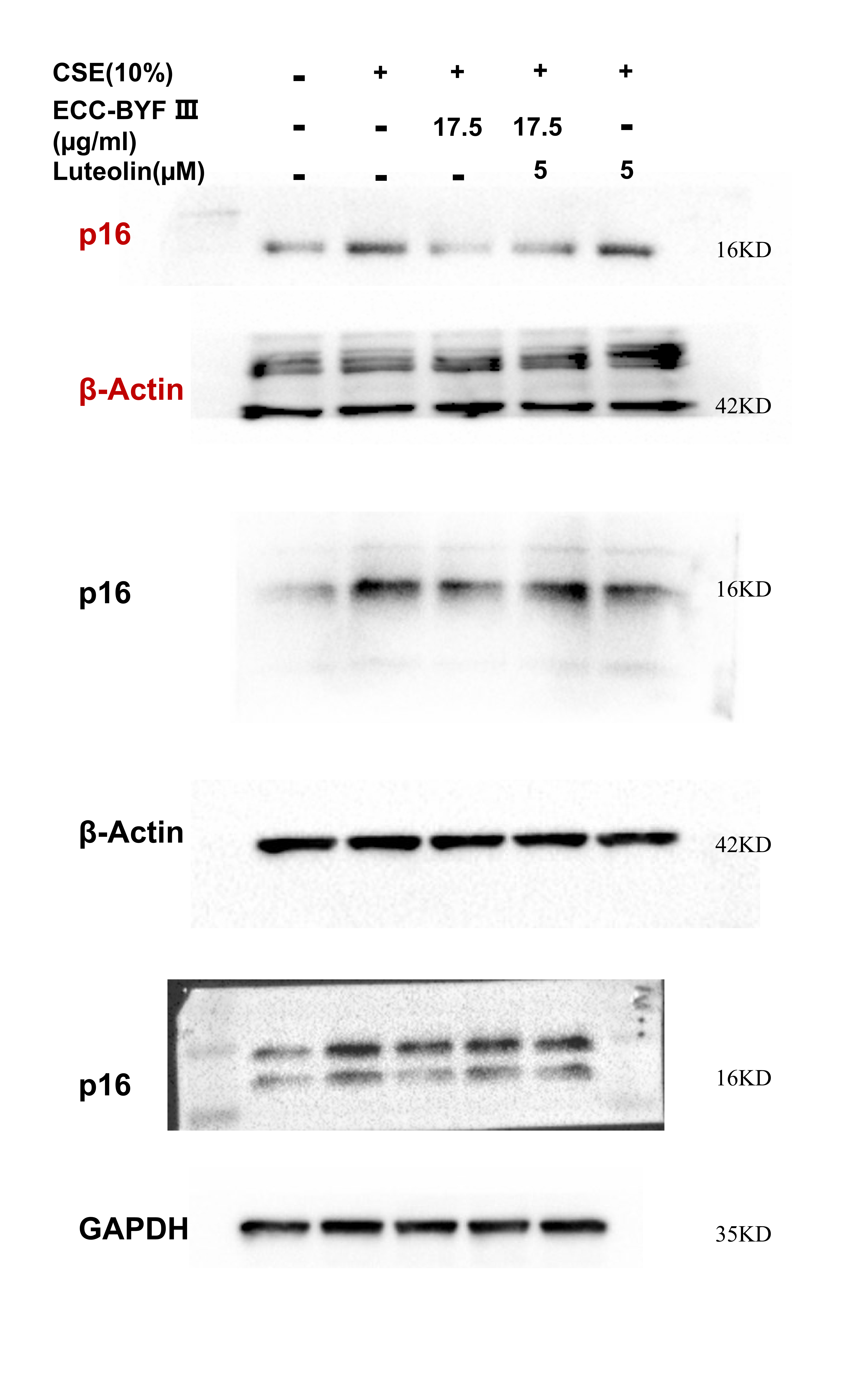

Supplement: Supplementary file 7 — Additional file 7. [file 12890_2022_2191_MOESM7_ESM.zip › Digital imange figure 16.png]

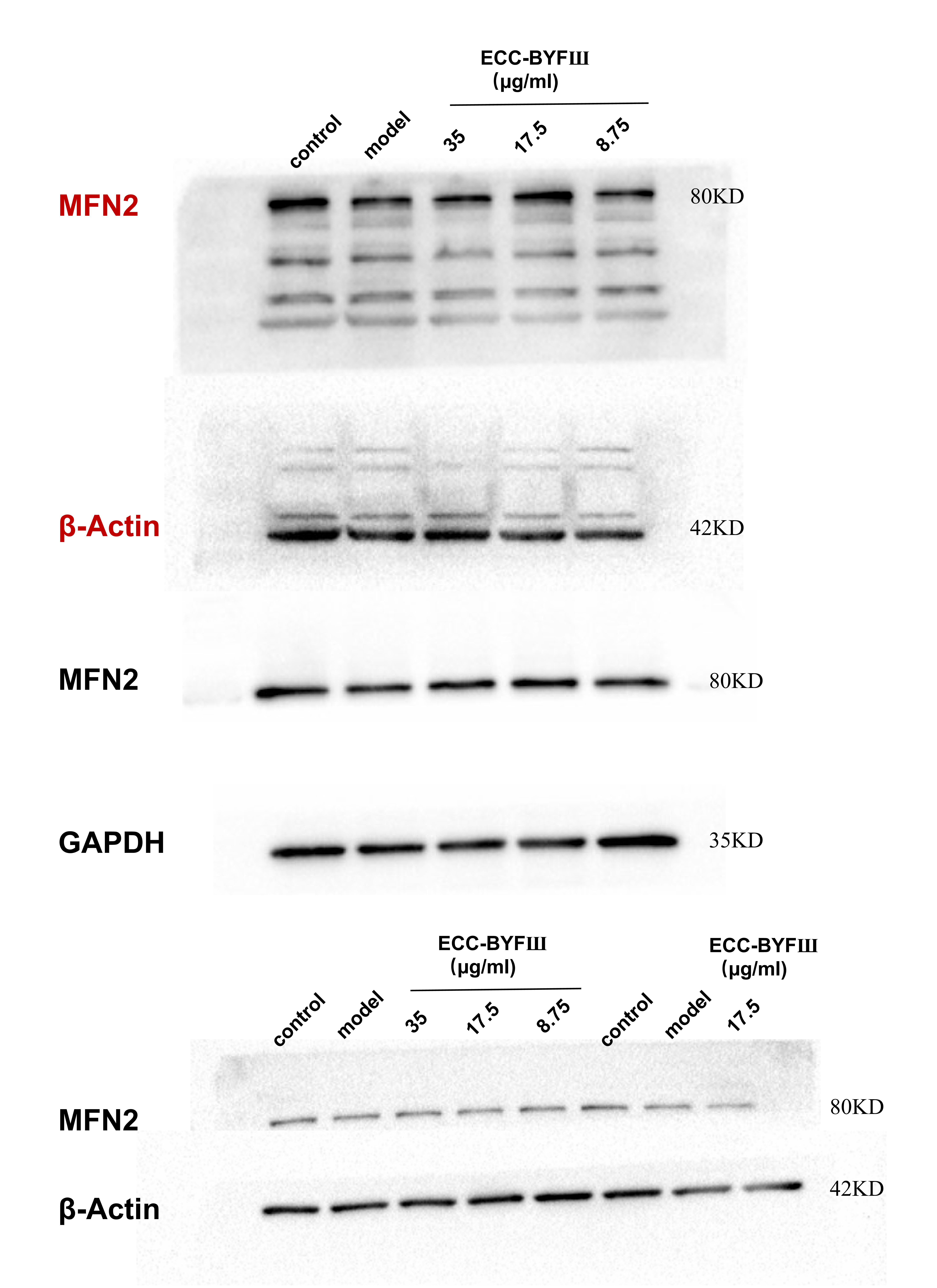

Supplement: Supplementary file 7 — Additional file 7. [file 12890_2022_2191_MOESM7_ESM.zip › Digital imange figure 5.png]

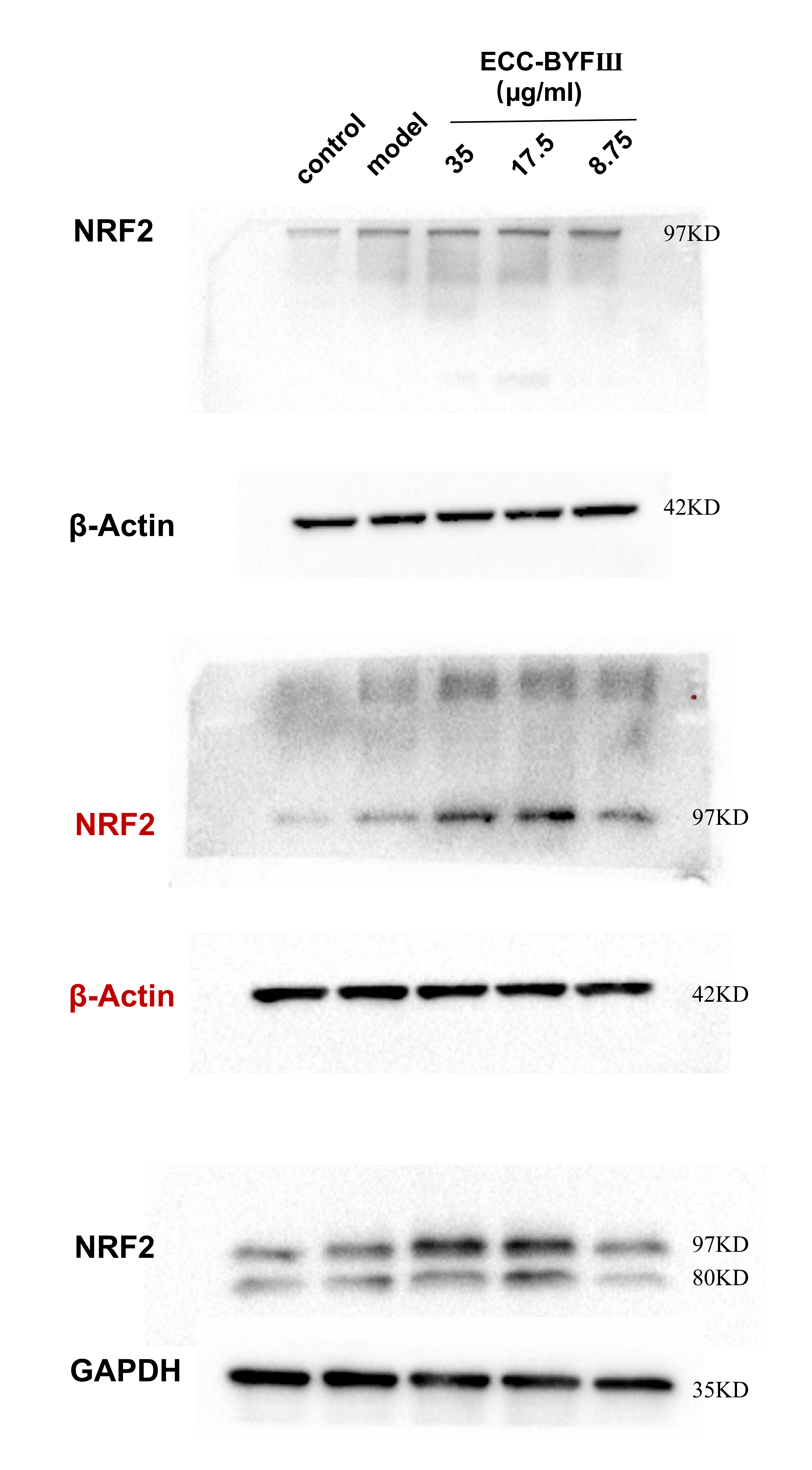

Supplement: Supplementary file 7 — Additional file 7. [file 12890_2022_2191_MOESM7_ESM.zip › Digital imange figure 7.png]

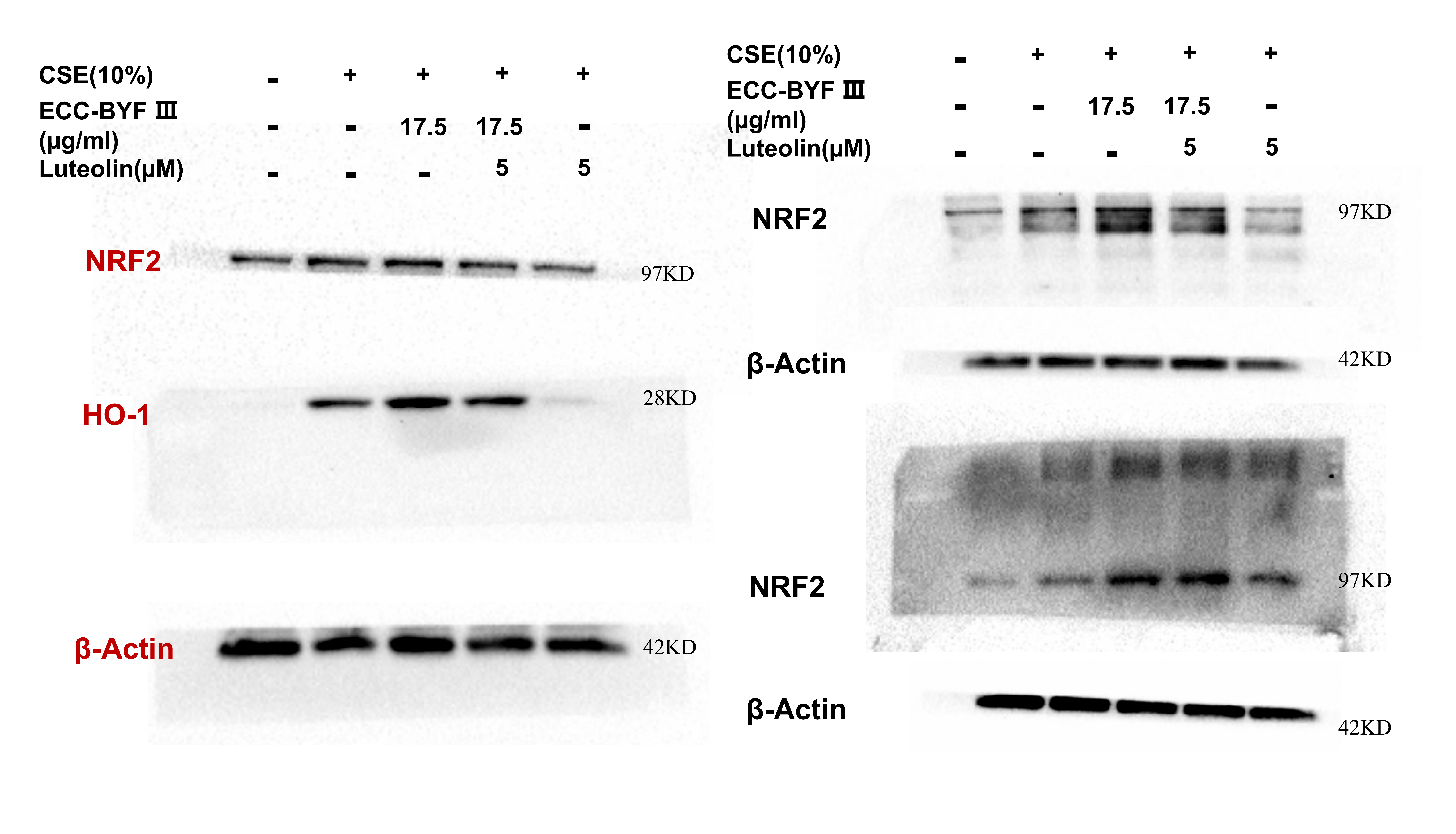

Supplement: Supplementary file 7 — Additional file 7. [file 12890_2022_2191_MOESM7_ESM.zip › Digital imange figure 9.png]
