# Supplementary material for: Effective-component compatibility of Bufei Yishen formula III ameliorated COPD by improving airway epithelial cell senescence by promoting mitophagy via the NRF2/PINK1 pathway
Source: BMC Pulm Med. 2022 Nov 22;22:434. doi: 10.1186/s12890-022-02191-9 (PMC9682796; doi:10.1186/s12890-022-02191-9)
Supplement: Supplementary file 7 — Additional file 7. [file 12890_2022_2191_MOESM7_ESM.zip › Digital image integrity.pdf]

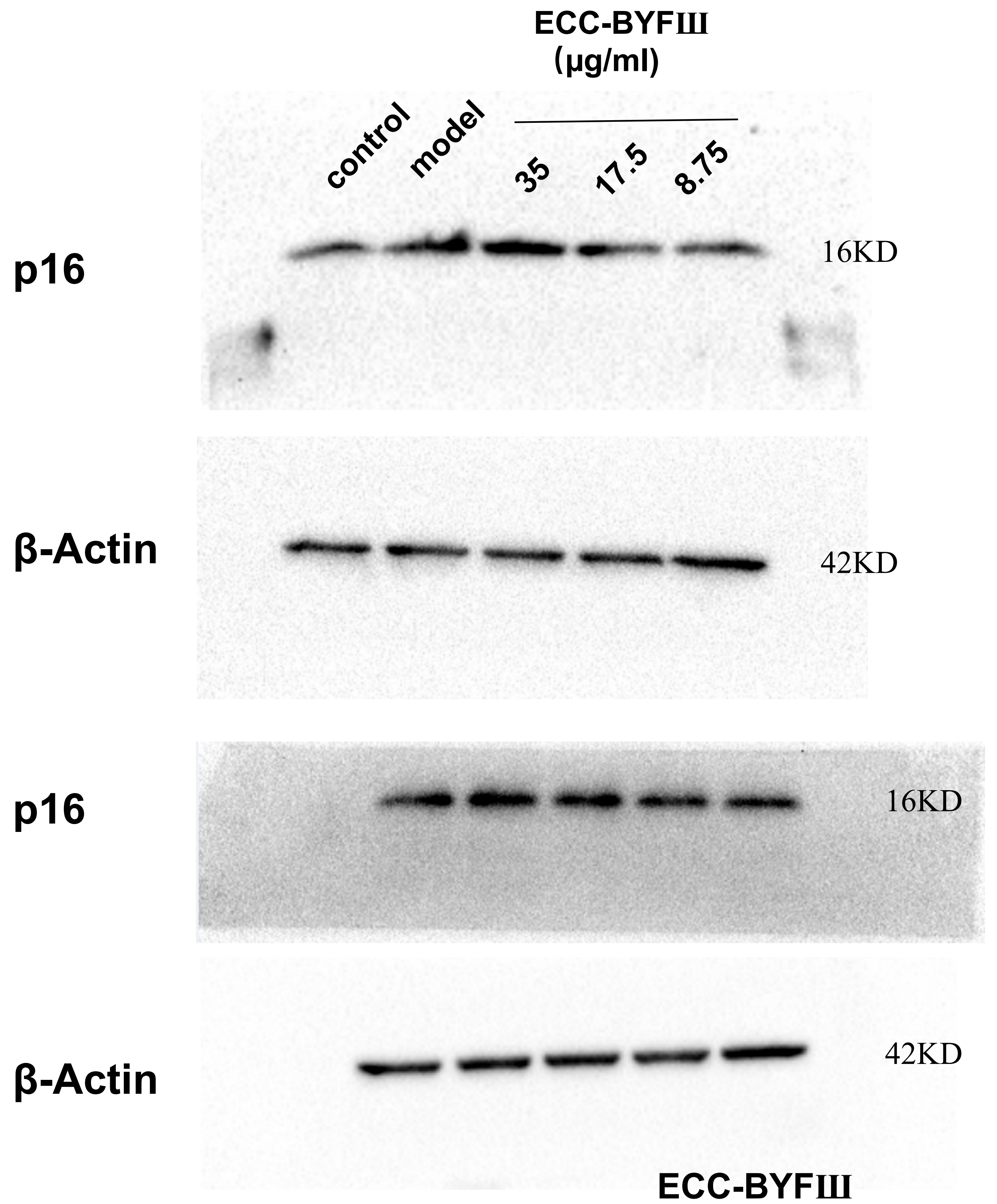
ECC-BYFⅢ
(μg/ml)
control
model
35
17.5
8.75
p16
16KD
β-Actin
42KD
p16
16KD
β-Actin
42KD

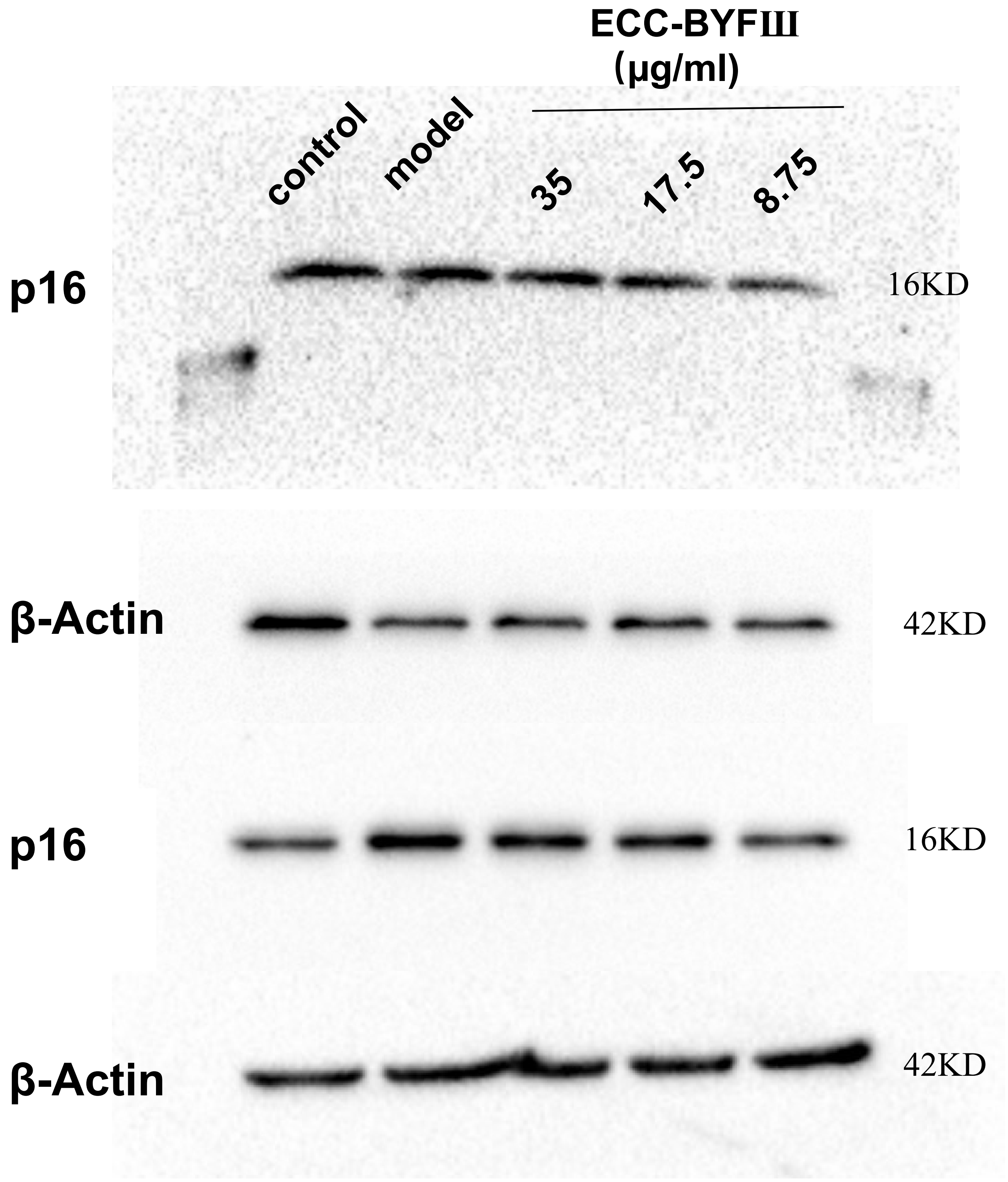
ECC-BYFⅢ
(μg/ml)
control
model
35
17.5
8.75
p16
16KD
β-Actin
42KD
p16
16KD
β-Actin
42KD

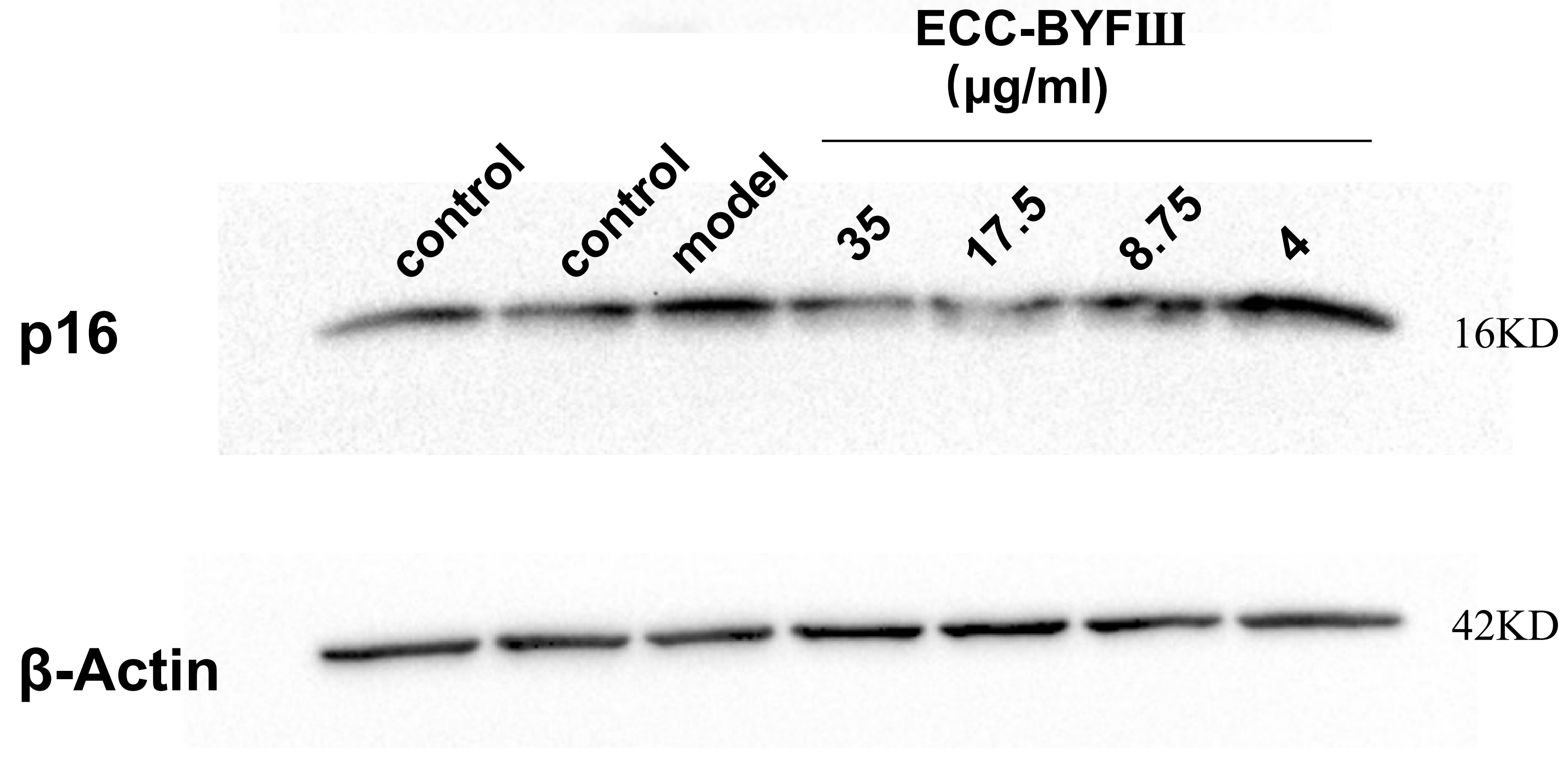
ECC-BYFⅢ
(μg/ml)
control
control
model
35
17.5
8.75
4
p16
16KD
β-Actin
42KD

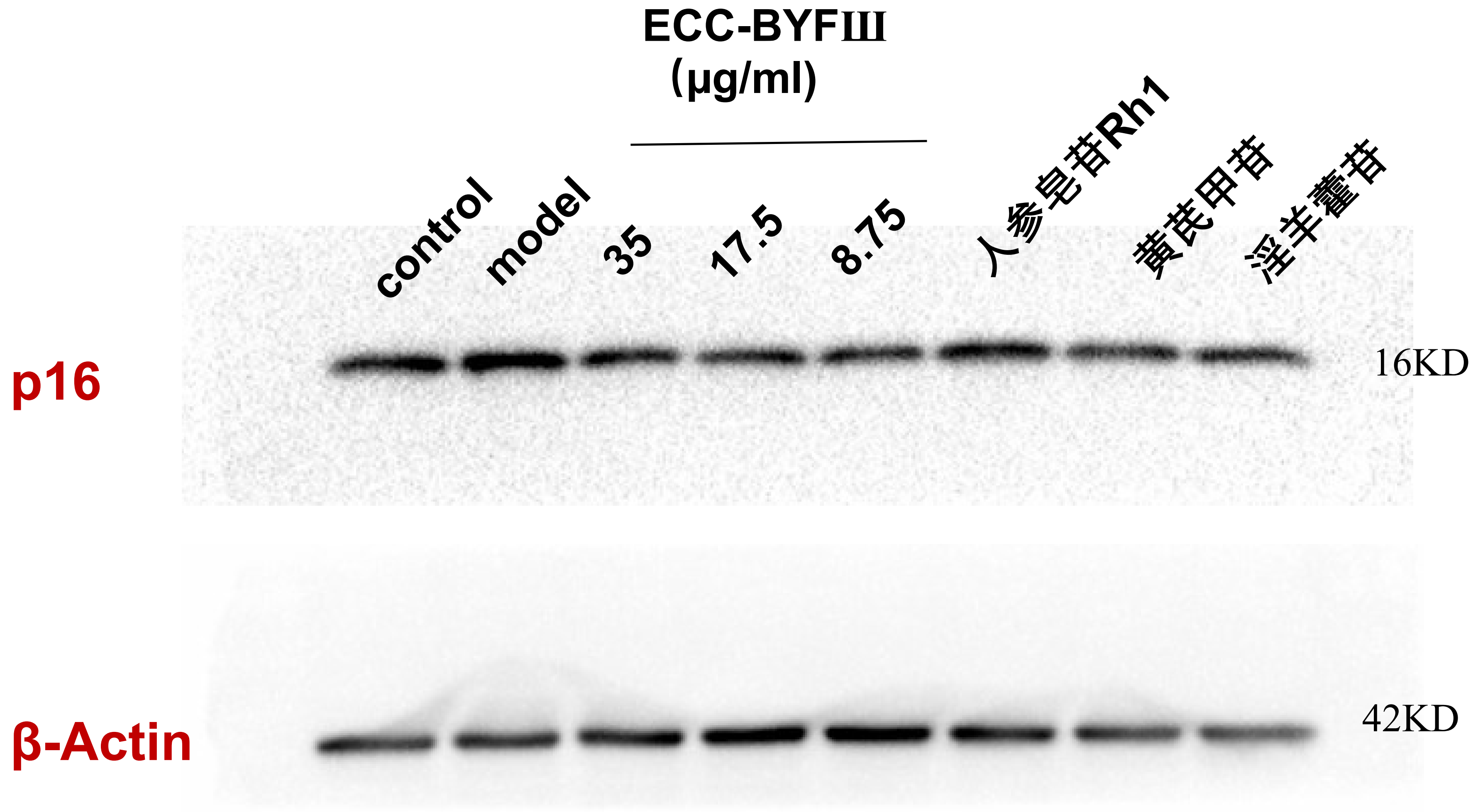
ECC-BYFⅢ
(μg/ml)
control
model
35
17.5
8.75
人参皂苷Rh1
黄芪甲苷
淫羊藿苷
p16
16KD
β-Actin
42KD

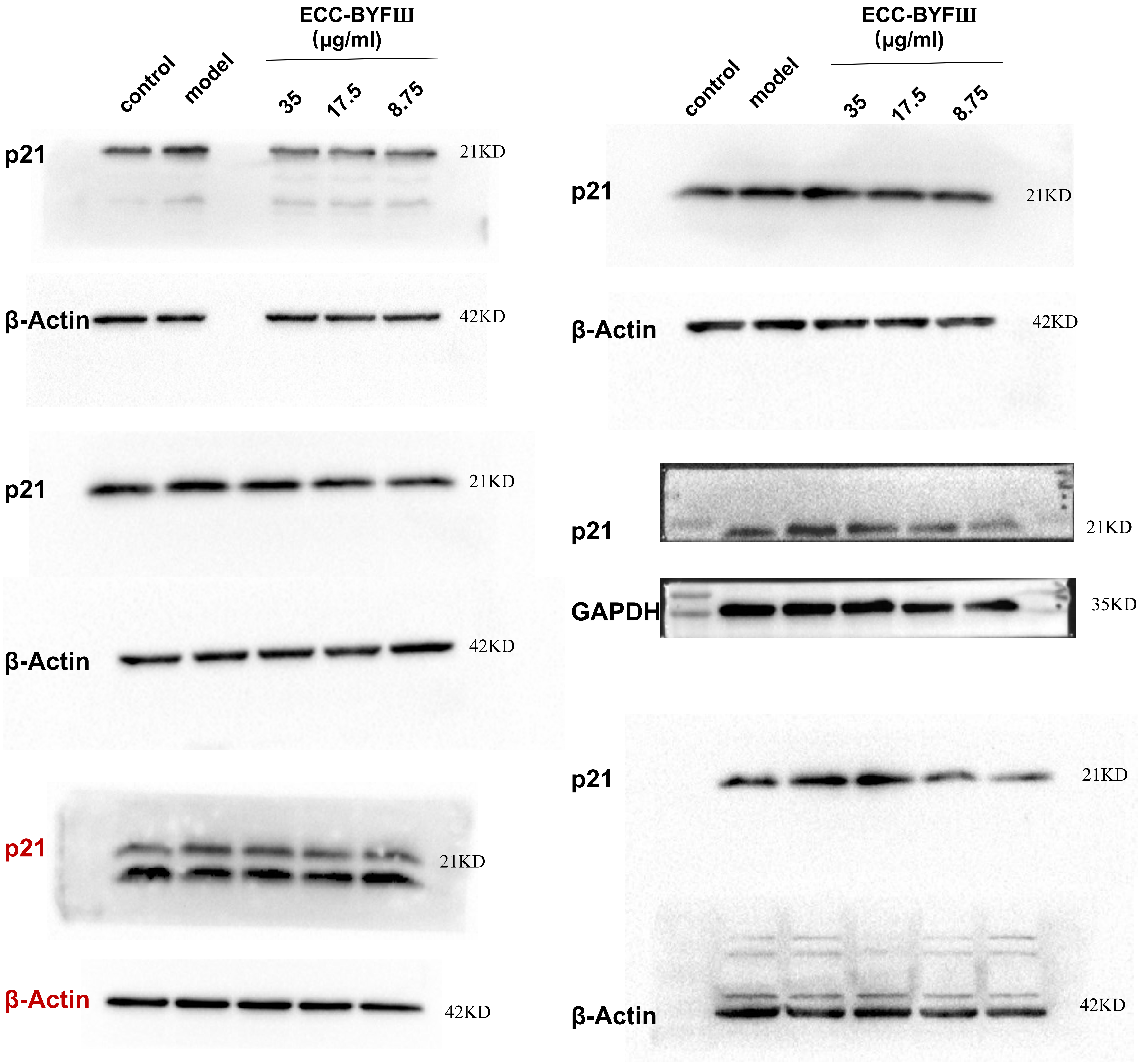
ECC-BYFIII (μg/ml)
control
model
35
17.5
8.75
p21
21KD
β-Actin
42KD
p21
21KD
β-Actin
42KD
p21
21KD
β-Actin
42KD
ECC-BYFIII (μg/ml)
control
model
35
17.5
8.75
p21
21KD
β-Actin
42KD
p21
21KD
GAPDH
35KD
p21
21KD
β-Actin
42KD

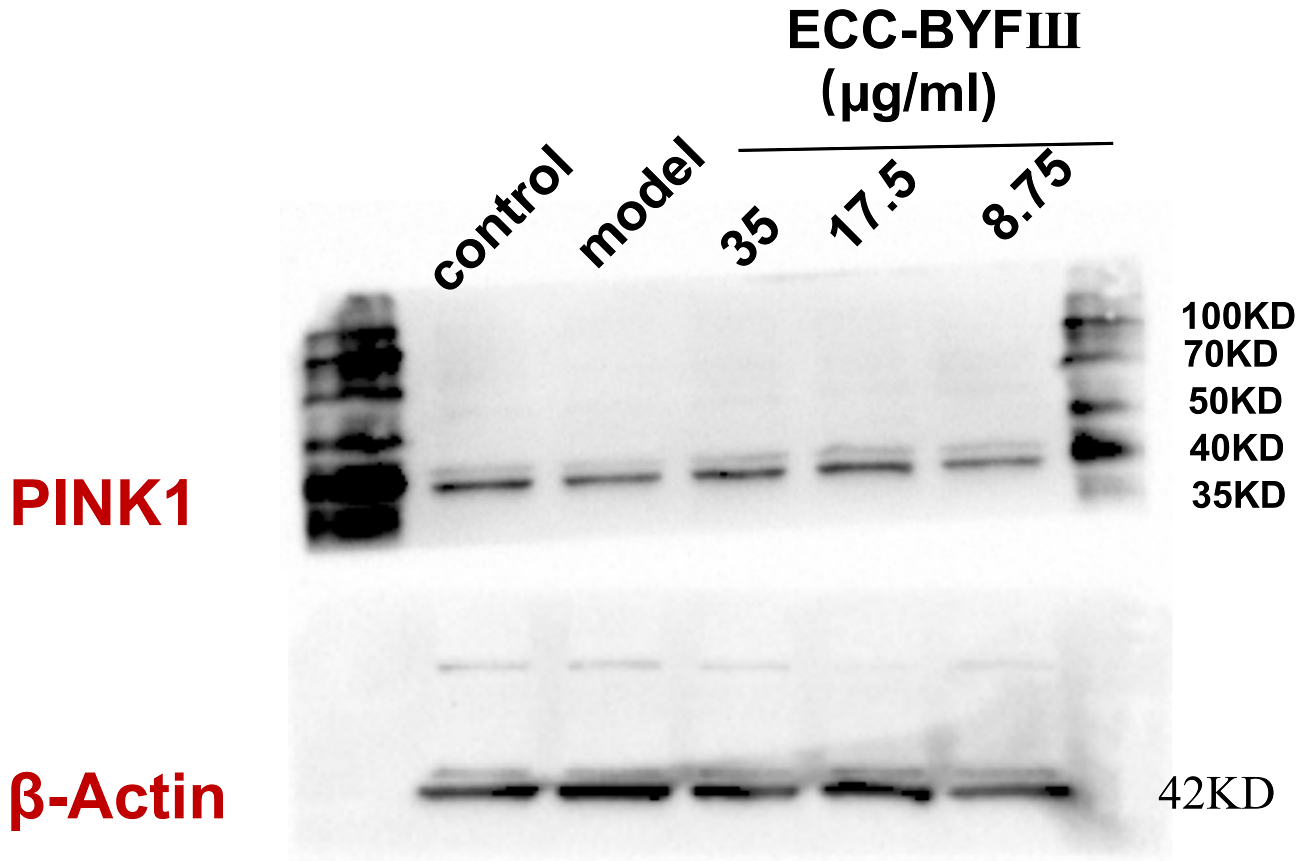
ECC-BYFⅢ (μg/ml)
control
model
35
17.5
8.75
100KD
70KD
50KD
40KD
35KD
PINK1
β-Actin
42KD

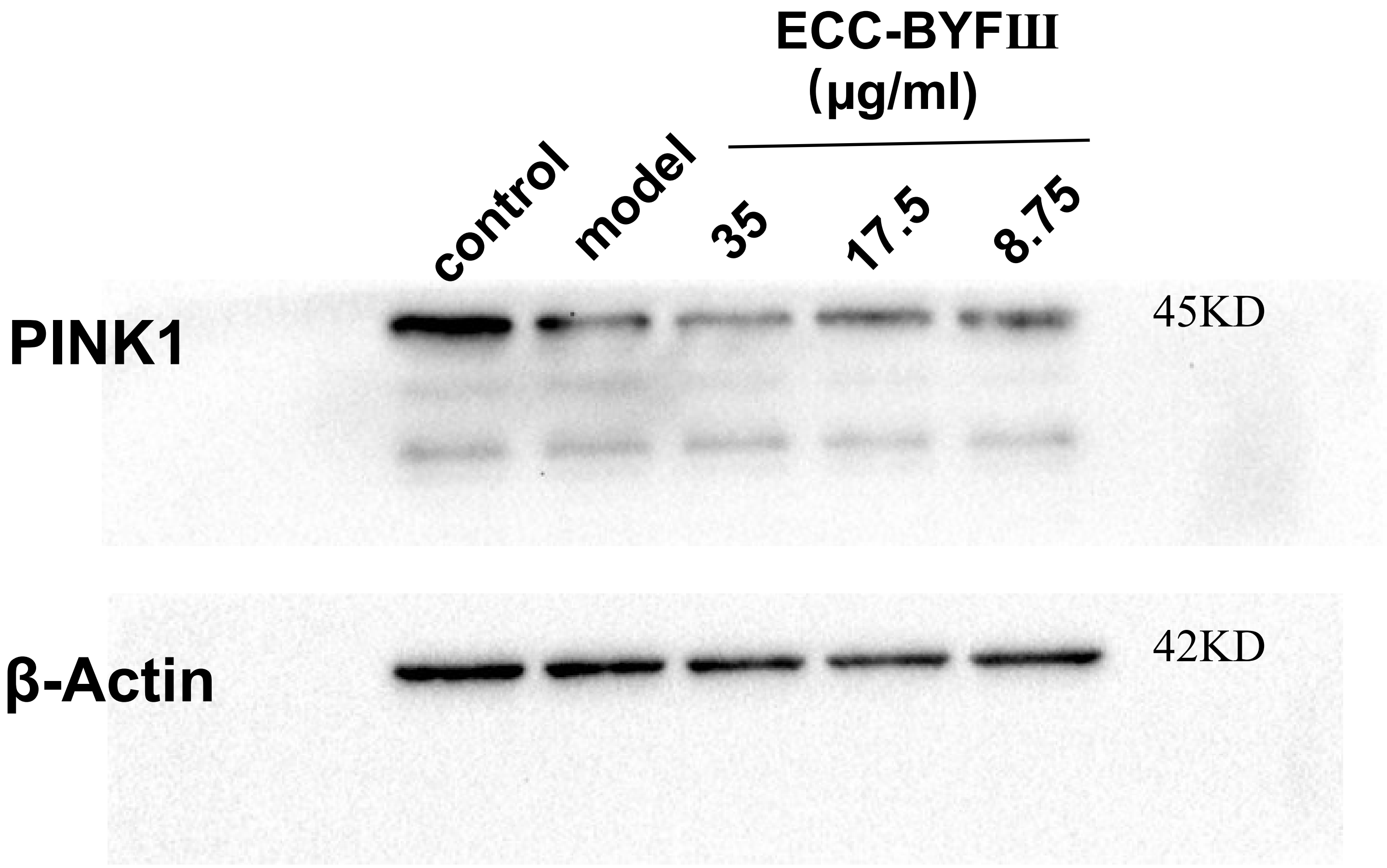
ECC-BYFⅢ (μg/ml)
control
model
35
17.5
8.75
PINK1
45KD
β-Actin
42KD

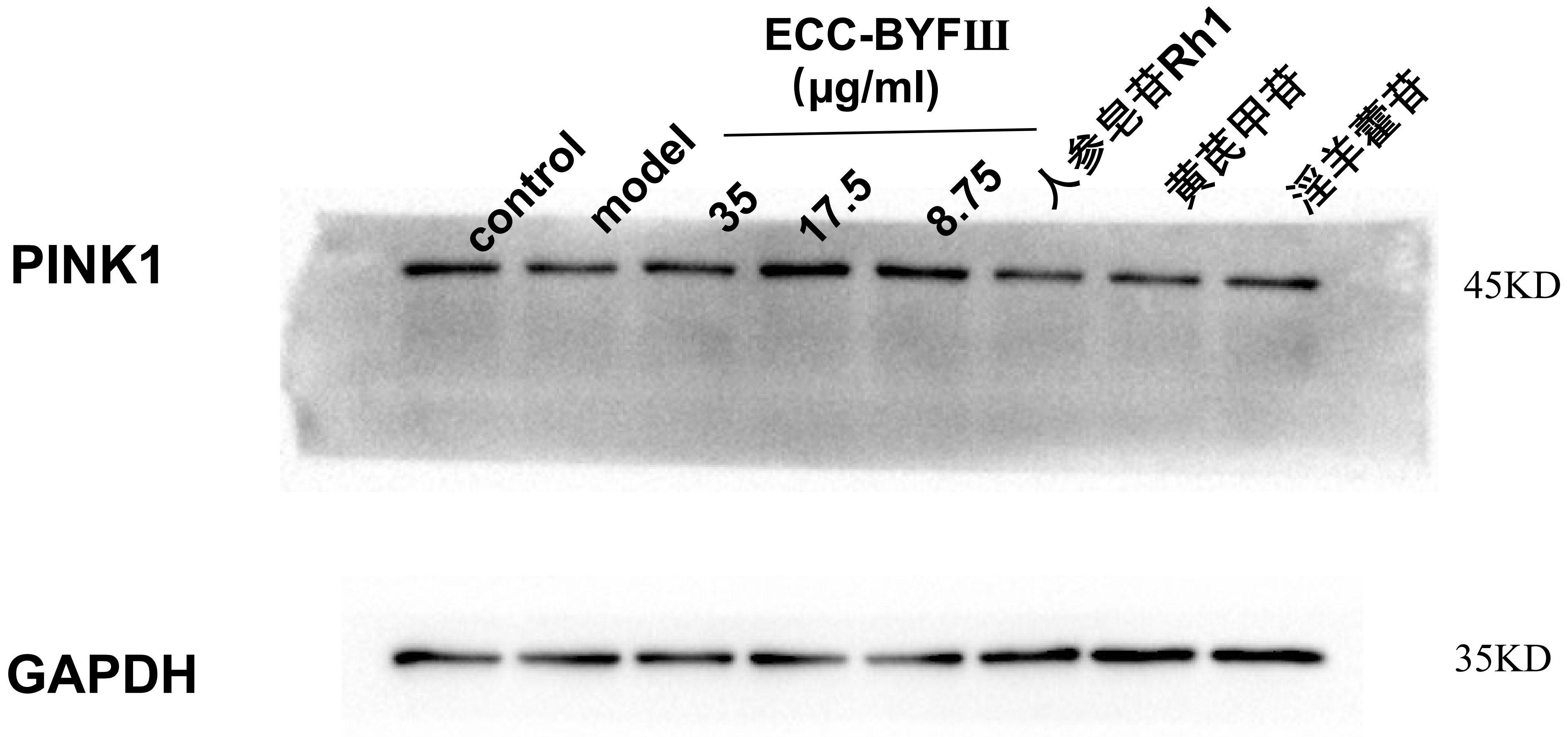
ECC-BYFⅢ (μg/ml)
control
model
35
17.5
8.75
人参皂苷Rh1
黄芪甲苷
淫羊藿苷
PINK1
45KD
GAPDH
35KD

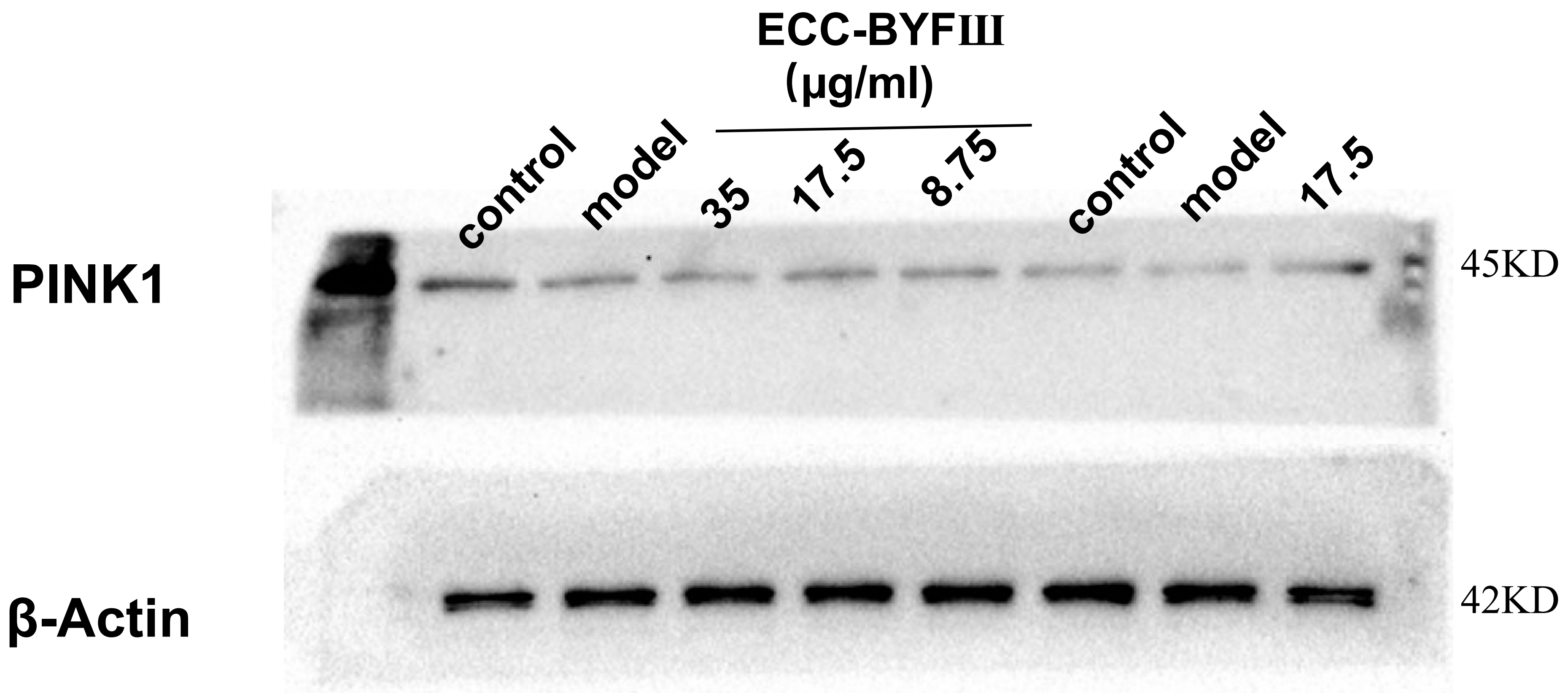
ECC-BYFⅢ (μg/ml)
control
model
35
17.5
8.75
control
model
17.5
PINK1
45KD
β-Actin
42KD

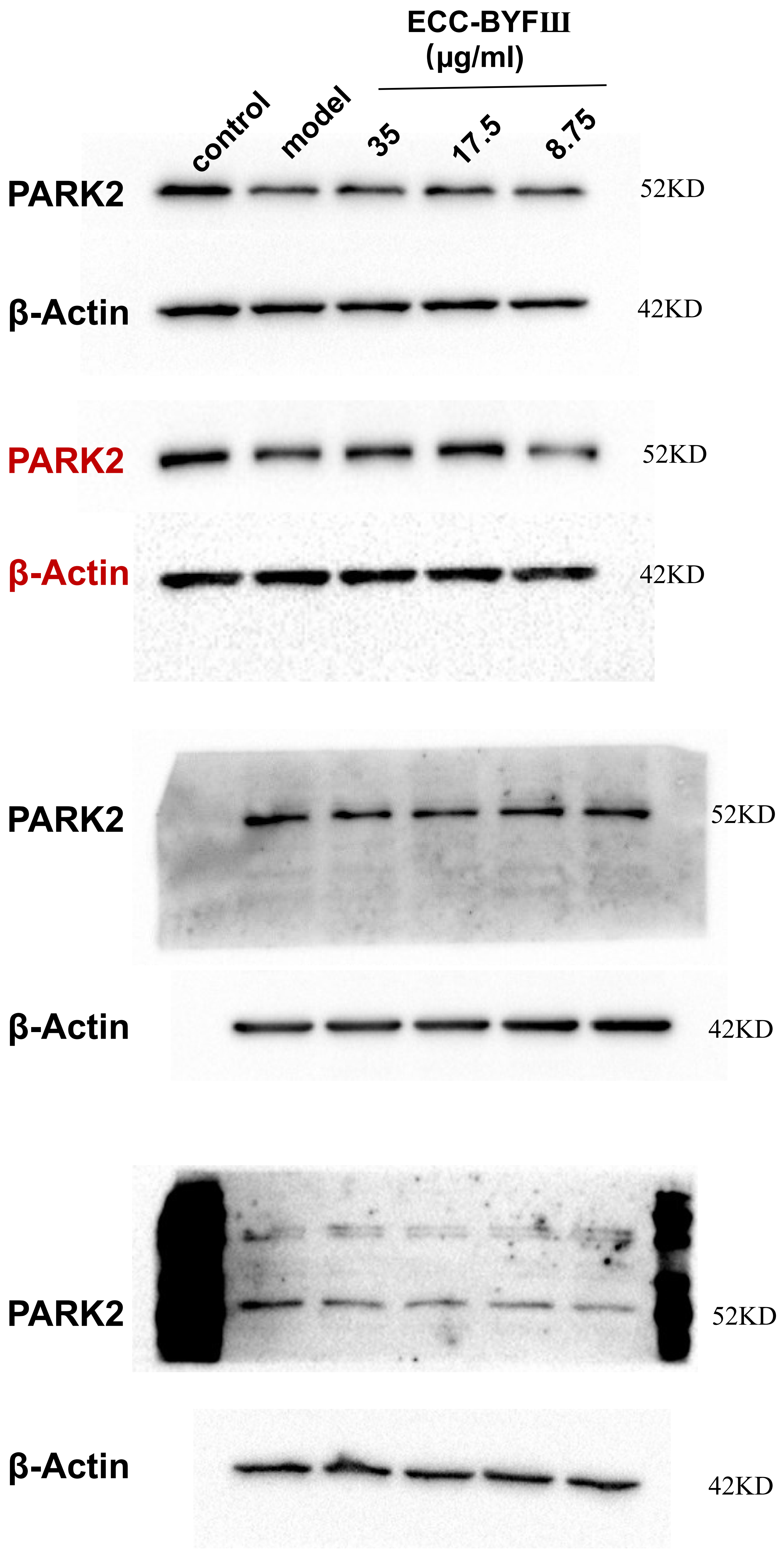
ECC-BYFШ
(μg/ml)
control
model
35
17.5
8.75
PARK2
52KD
β-Actin
42KD
PARK2
52KD
β-Actin
42KD
PARK2
52KD
β-Actin
42KD
PARK2
52KD
β-Actin
42KD

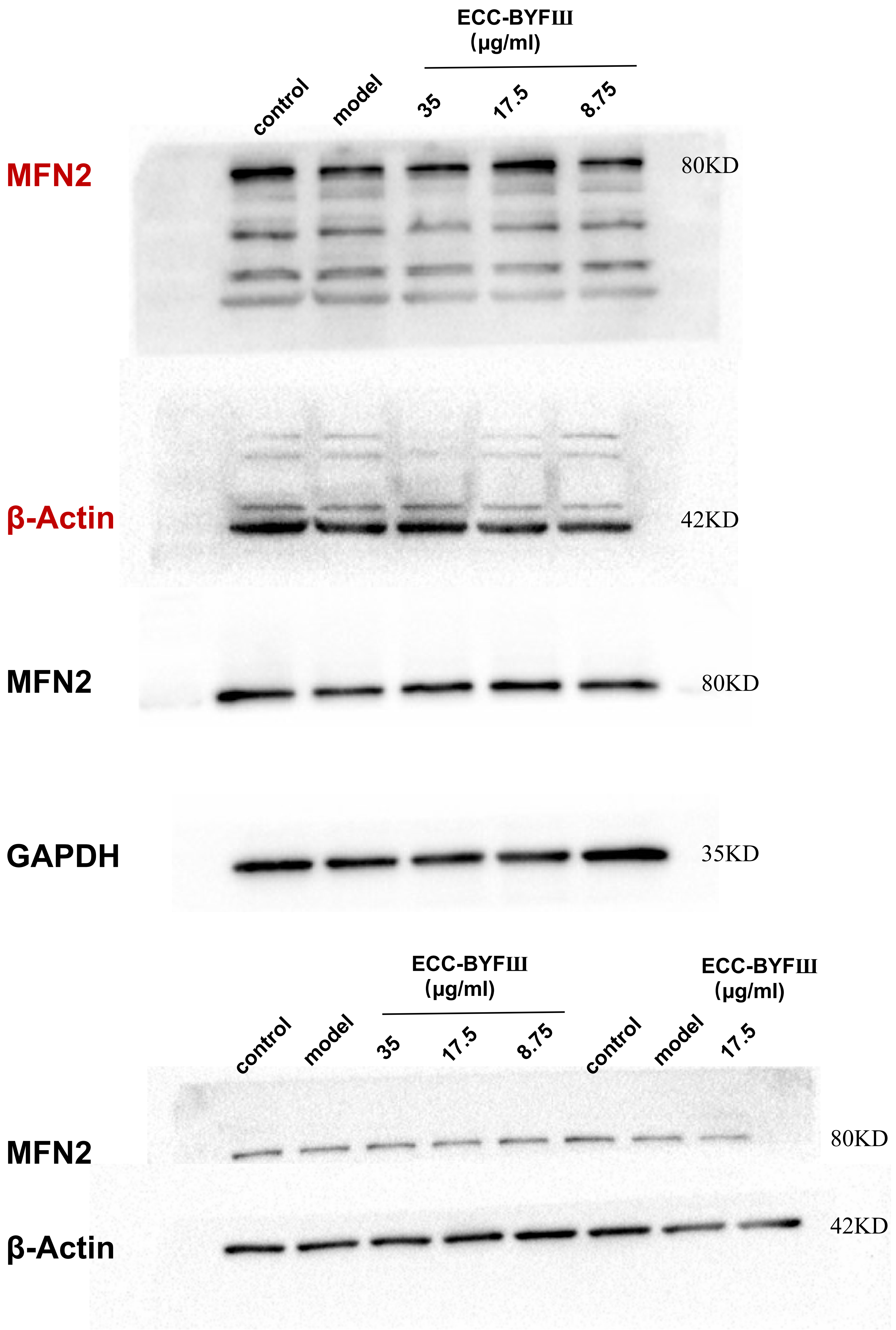
ECC-BYFIII
(μg/ml)
control
model
35
17.5
8.75
MFN2
80KD
β-Actin
42KD
MFN2
80KD
GAPDH
35KD
ECC-BYFIII
(μg/ml)
control
model
35
17.5
8.75
control
model
ECC-BYFIII
(μg/ml)
17.5
MFN2
80KD
β-Actin
42KD

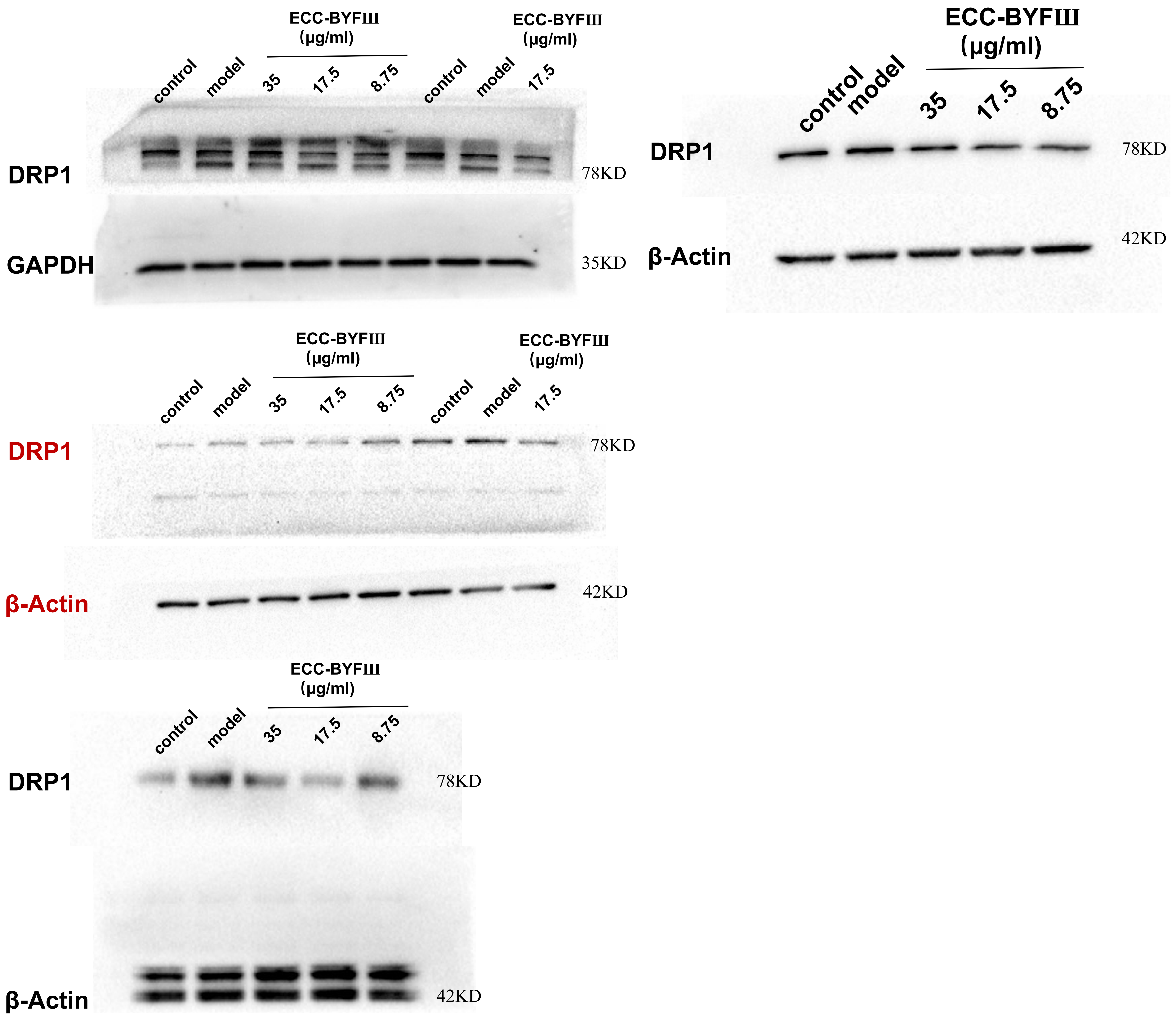
ECC-BYFШ (μg/ml)
control
model
35
17.5
8.75
control
model
17.5
ECC-BYFШ (μg/ml)
DRP1
78KD
GAPDH
35KD
ECC-BYFШ (μg/ml)
control
model
35
17.5
8.75
DRP1
78KD
β-Actin
42KD
ECC-BYFШ (μg/ml)
control
model
35
17.5
8.75
control
model
17.5
ECC-BYFШ (μg/ml)
DRP1
78KD
β-Actin
42KD
ECC-BYFШ (μg/ml)
control
model
35
17.5
8.75
DRP1
78KD
β-Actin
42KD

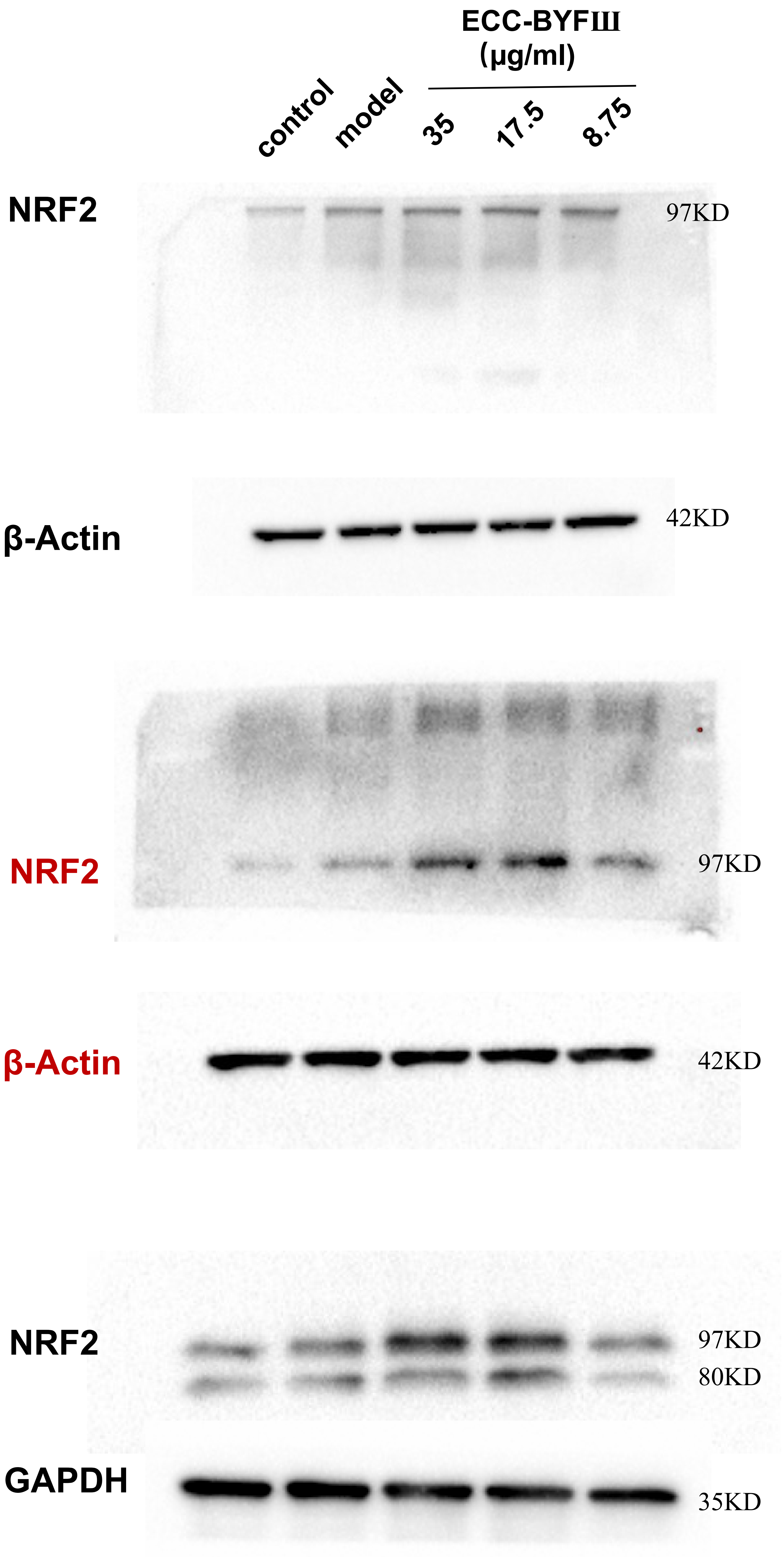

ECC-BYFⅢ
(μg/ml)
control
model
35
17.5
8.75
NRF2
97KD
β-Actin
42KD
NRF2
97KD
β-Actin
42KD
NRF2
97KD
80KD
GAPDH
35KD

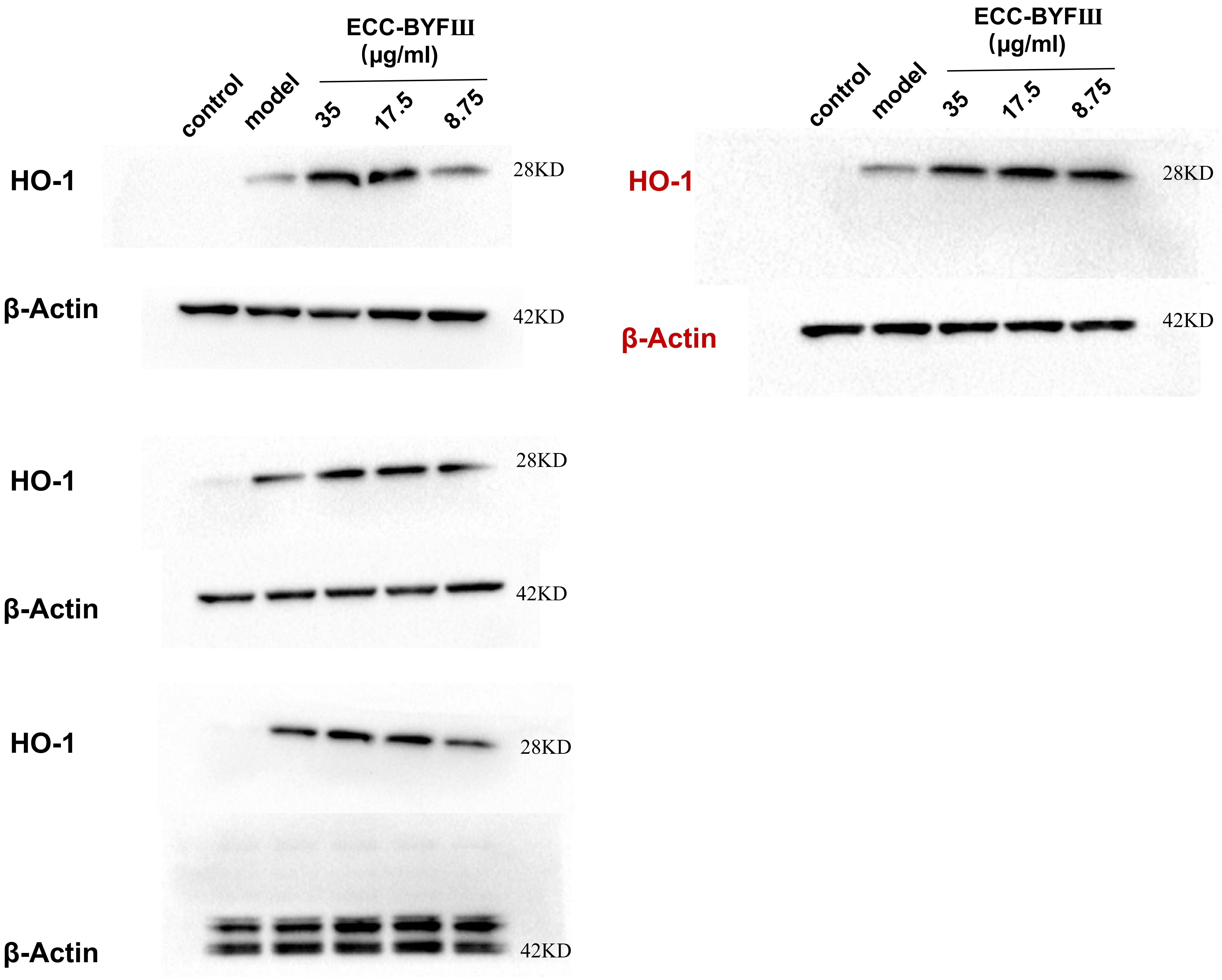
ECC-BYFⅢ
(μg/ml)
control
model
35
17.5
8.75
HO-1
28KD
β-Actin
42KD
HO-1
28KD
β-Actin
42KD
HO-1
28KD
β-Actin
42KD
ECC-BYFⅢ
(μg/ml)
control
model
35
17.5
8.75
HO-1
28KD
β-Actin
42KD

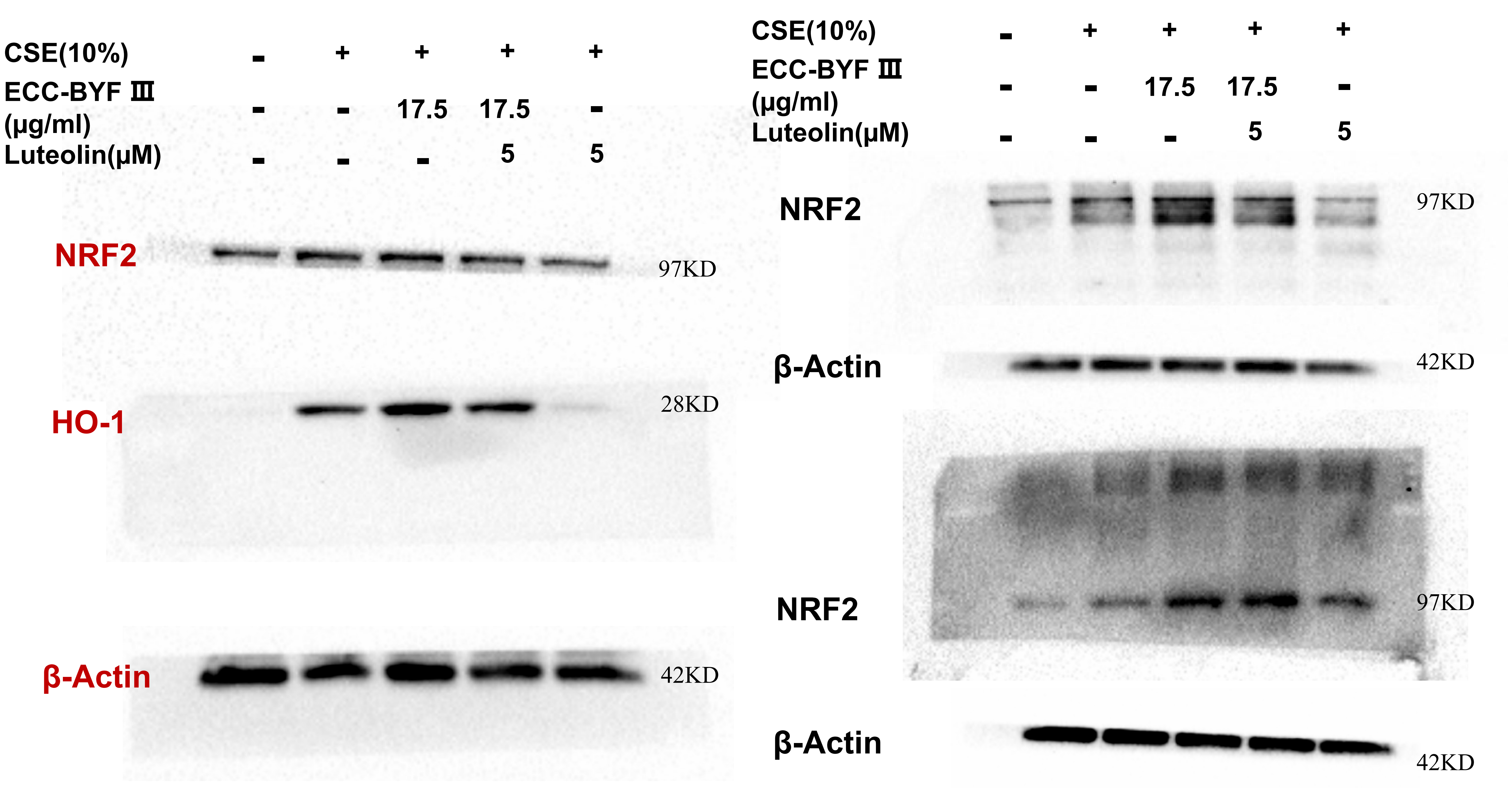
CSE(10%)
ECC-BYF Ⅲ (μg/ml)
Luteolin(μM)
- + + + +
- - 17.5 17.5 -
- - - 5 5
NRF2
97KD
HO-1
28KD
β-Actin
42KD
CSE(10%)
ECC-BYF Ⅲ (μg/ml)
Luteolin(μM)
- + + + +
- - 17.5 17.5 -
- - - 5 5
NRF2
97KD
β-Actin
42KD
NRF2
97KD
β-Actin
42KD

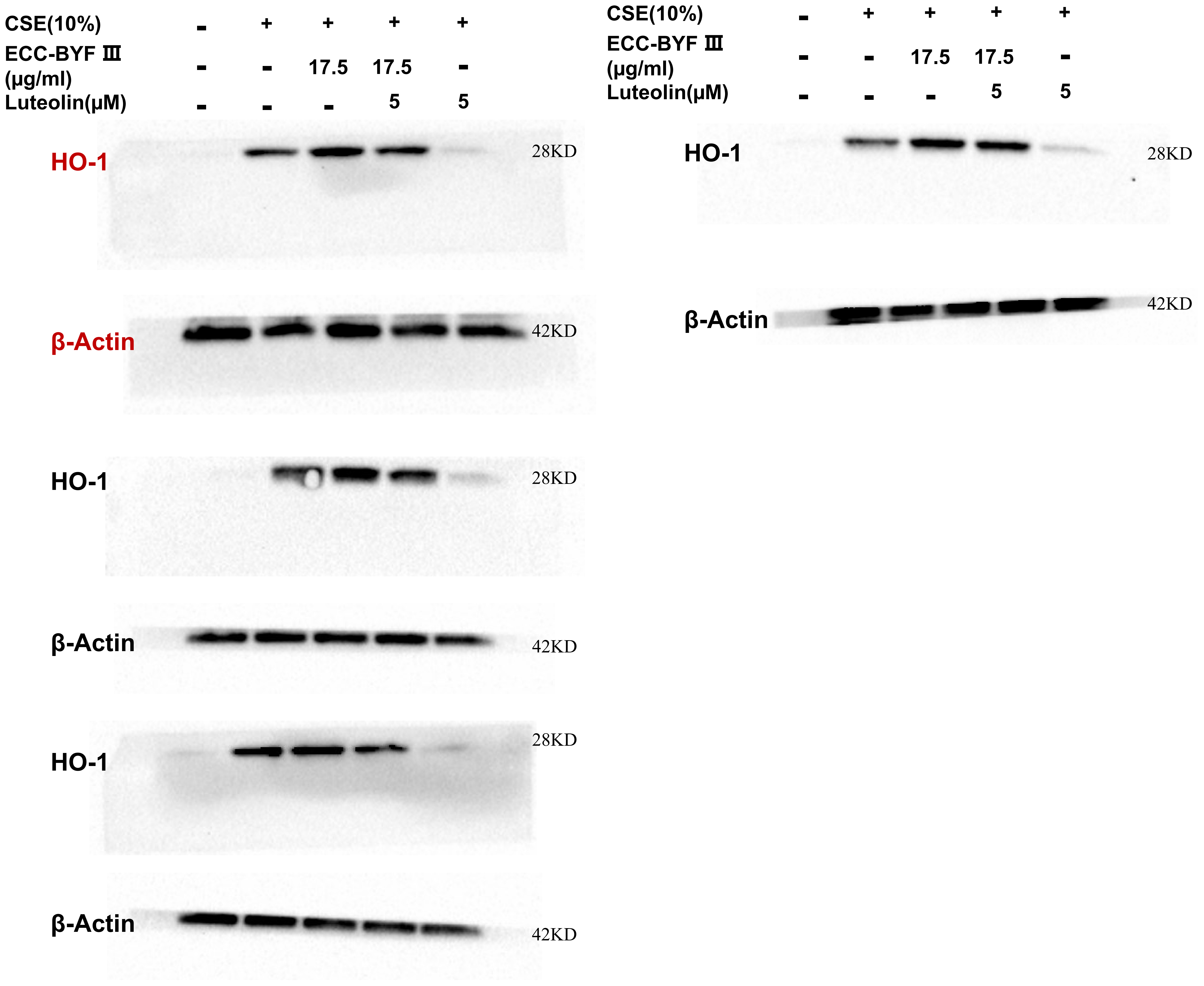
CSE(10%)
ECC-BYF Ⅲ (μg/ml)
Luteolin(μM)
17.5
5
HO-1
28KD
β-Actin
42KD

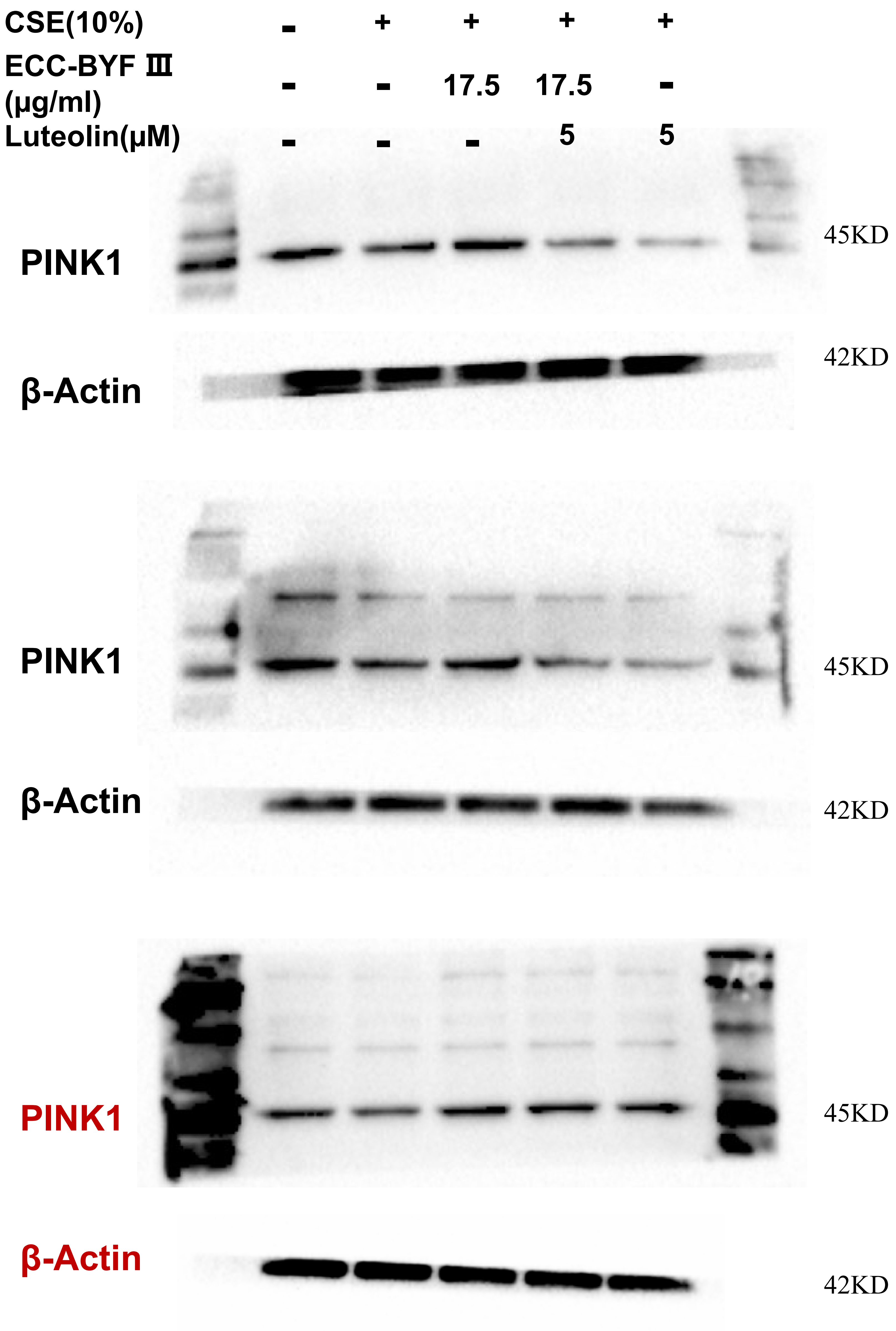
CSE(10%)
ECC-BYF Ⅲ (μg/ml)
Luteolin(μM)
17.5
17.5
5
5
PINK1
45KD
β-Actin
42KD
PINK1
45KD
β-Actin
42KD
PINK1
45KD
β-Actin
42KD

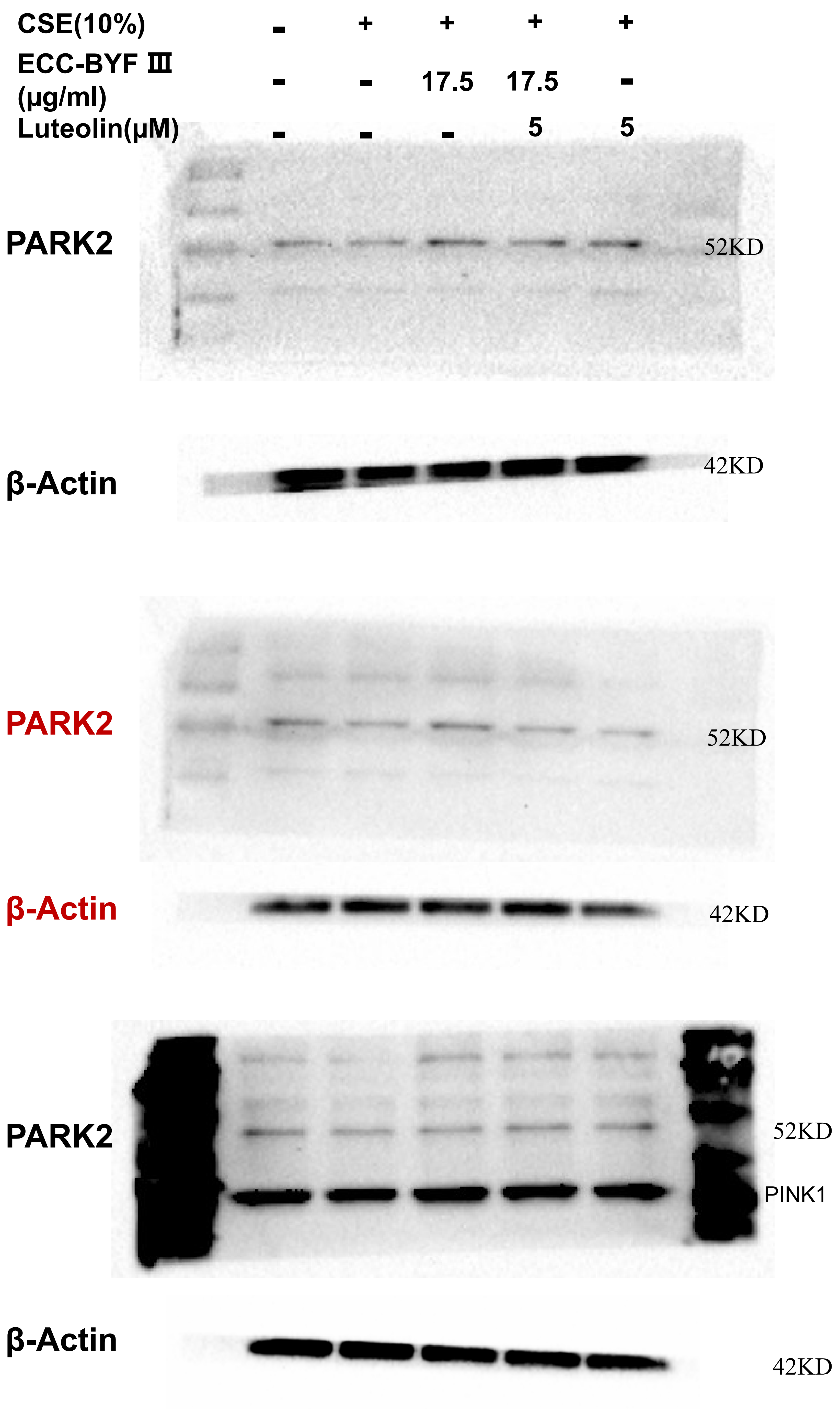
CSE(10%) - + + + +
ECC-BYF Ⅲ (μg/ml) - - 17.5 17.5 -
Luteolin(μM) - - - 5 5
PARK2
52KD
β-Actin
42KD
PARK2
52KD
β-Actin
42KD
PARK2
52KD
PINK1
β-Actin
42KD

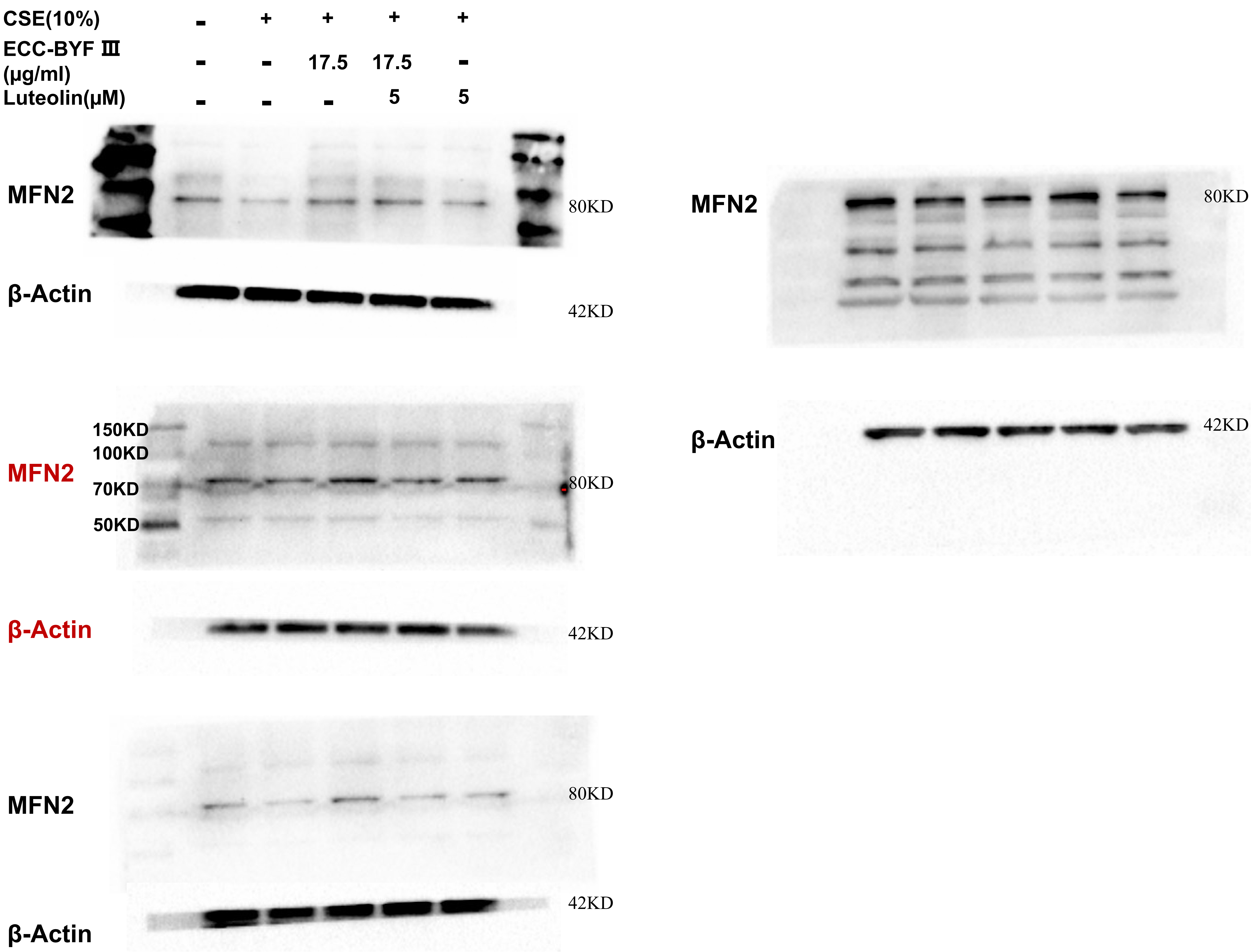
CSE(10%) - + + + +
ECC-BYF Ⅲ (μg/ml) - - 17.5 17.5 -
Luteolin(μM) - - - 5 5
MFN2
80KD
β-Actin
42KD
150KD
100KD
MFN2
70KD
50KD
80KD
β-Actin
42KD
MFN2
80KD
42KD
β-Actin
MFN2
80KD
β-Actin
42KD

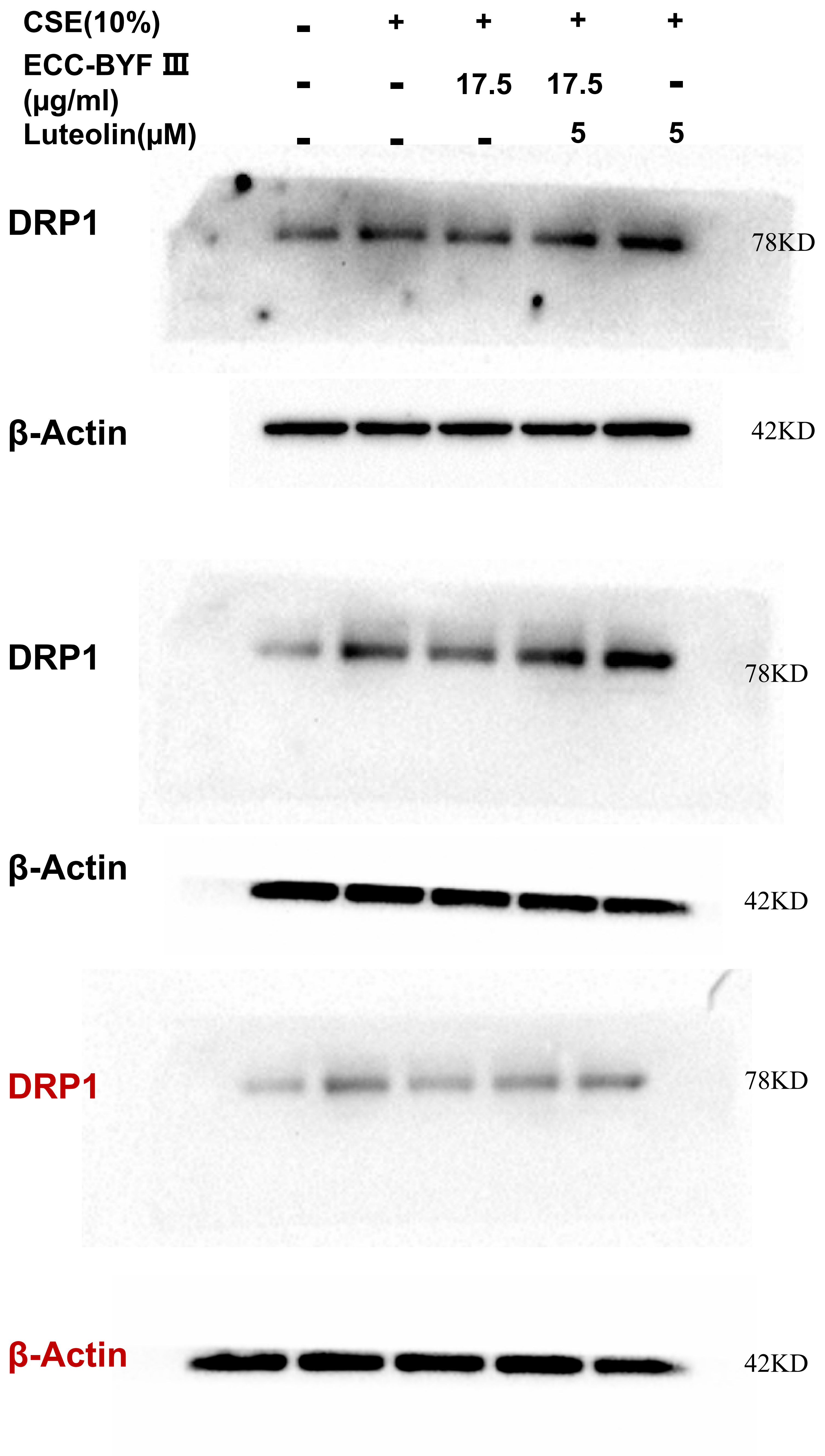
CSE(10%) - + + + +
ECC-BYF Ⅲ (μg/ml) - - 17.5 17.5 -
Luteolin(μM) - - - 5 5
DRP1
78KD
β-Actin
42KD
DRP1
78KD
β-Actin
42KD
DRP1
78KD
β-Actin
42KD

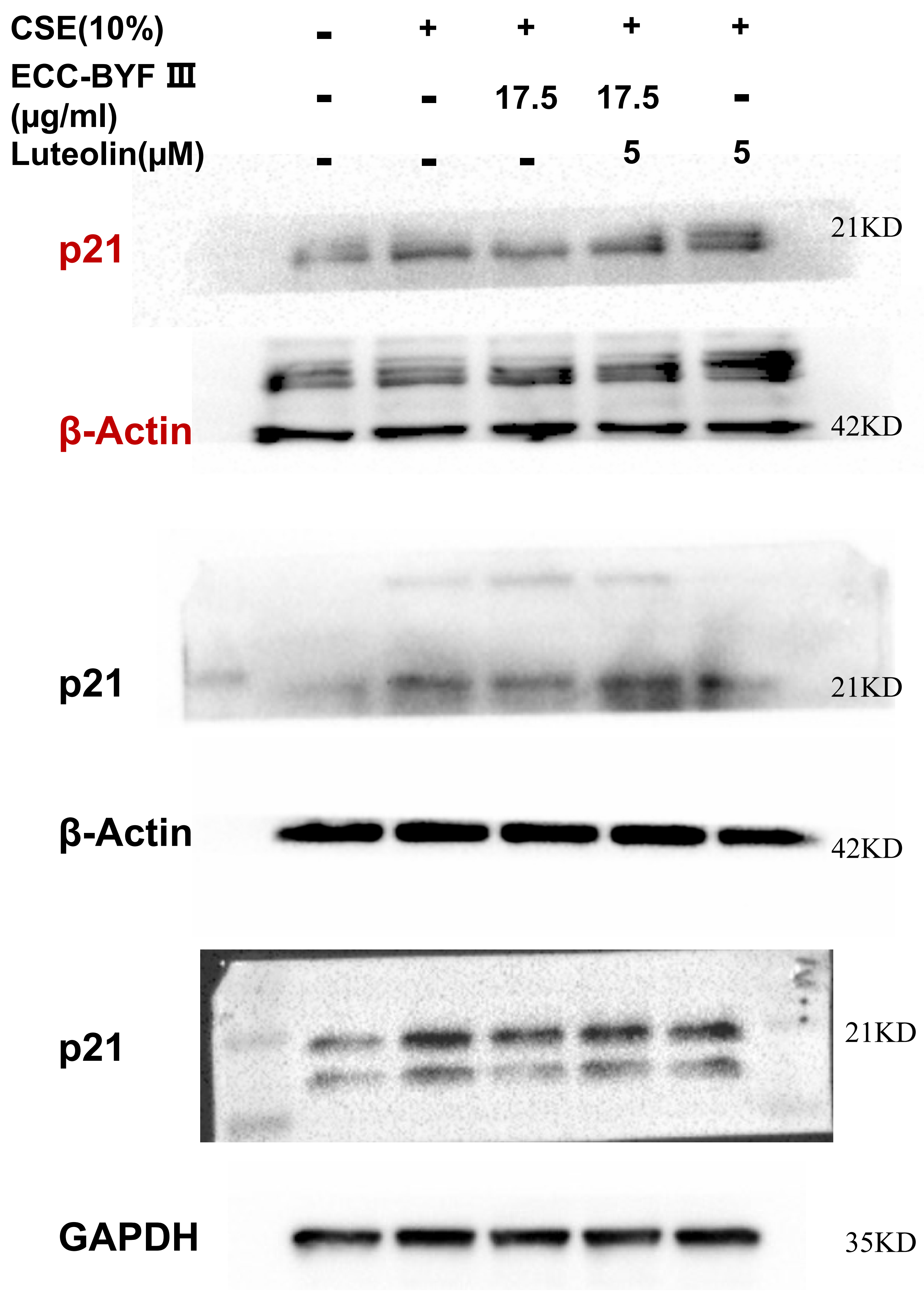
CSE(10%) - + + + +
ECC-BYF Ⅲ (μg/ml) - - 17.5 17.5 -
Luteolin(μM) - - - 5 5
p21
21KD
β-Actin
42KD
p21
21KD
β-Actin
42KD
p21
21KD
GAPDH
35KD

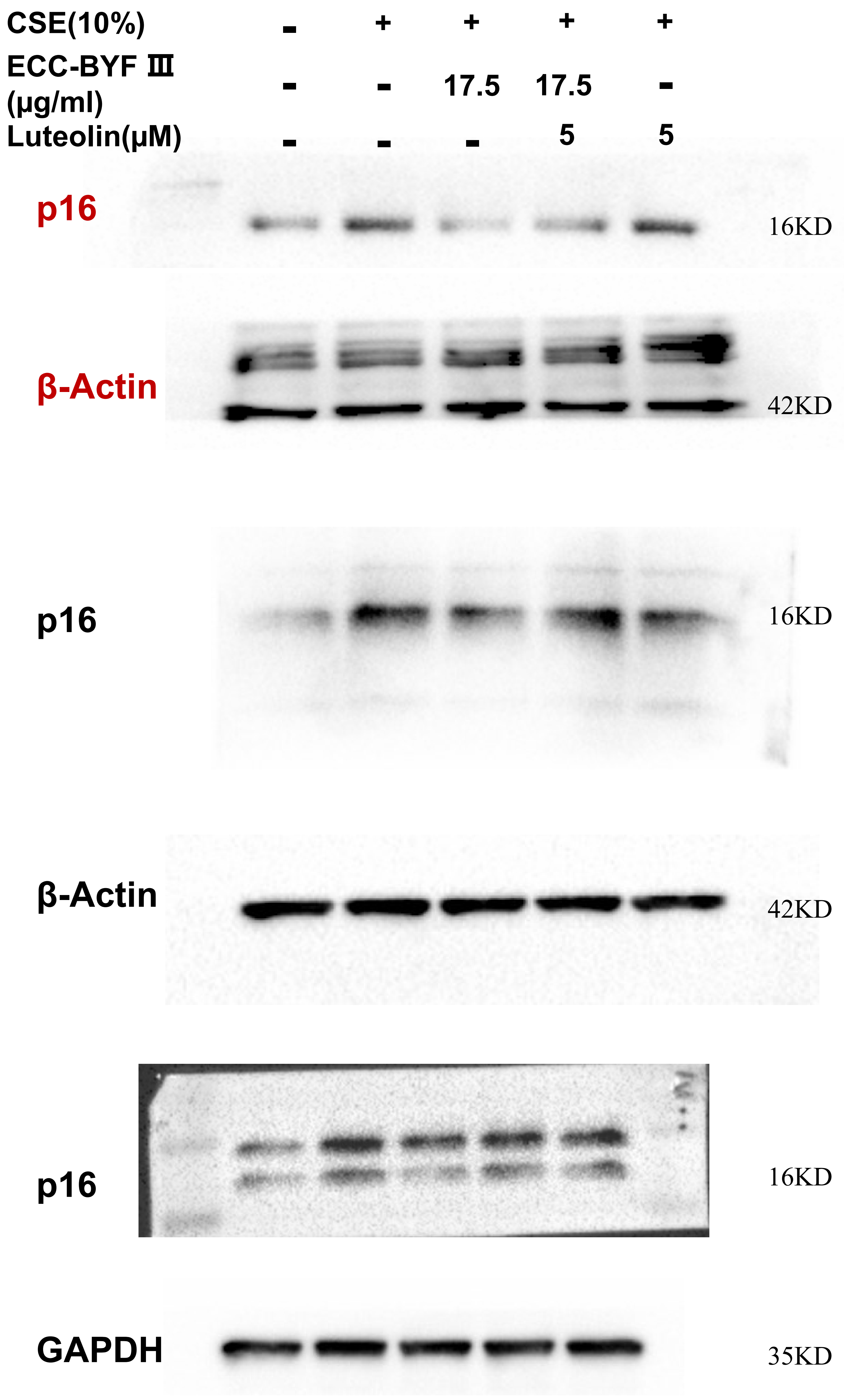
CSE(10%) - + + + +
ECC-BYF Ⅲ (μg/ml) - - 17.5 17.5 -
Luteolin(μM) - - - 5 5
p16
16KD
β-Actin
42KD
p16
16KD
β-Actin
42KD
p16
16KD
GAPDH
35KD
