# Supplementary material for: Effective-component compatibility of Bufei Yishen formula III ameliorated COPD by improving airway epithelial cell senescence by promoting mitophagy via the NRF2/PINK1 pathway
Source: BMC Pulm Med. 2022 Nov 22;22:434. doi: 10.1186/s12890-022-02191-9 (PMC9682796; doi:10.1186/s12890-022-02191-9)
Supplement: Supplementary file 7 — Additional file 7. [file 12890_2022_2191_MOESM7_ESM.zip › original image.pdf]

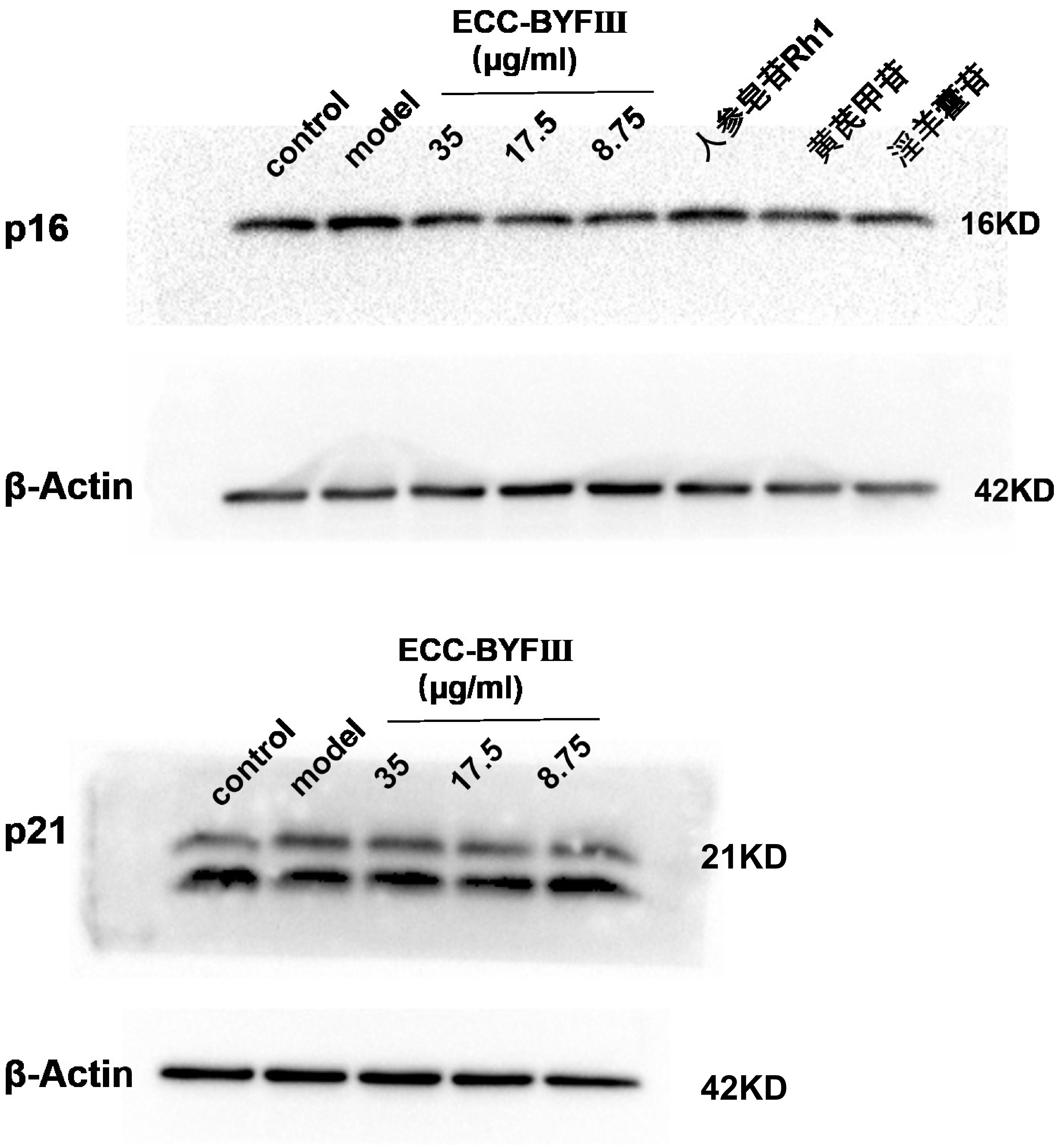

Supplementary Figure 1

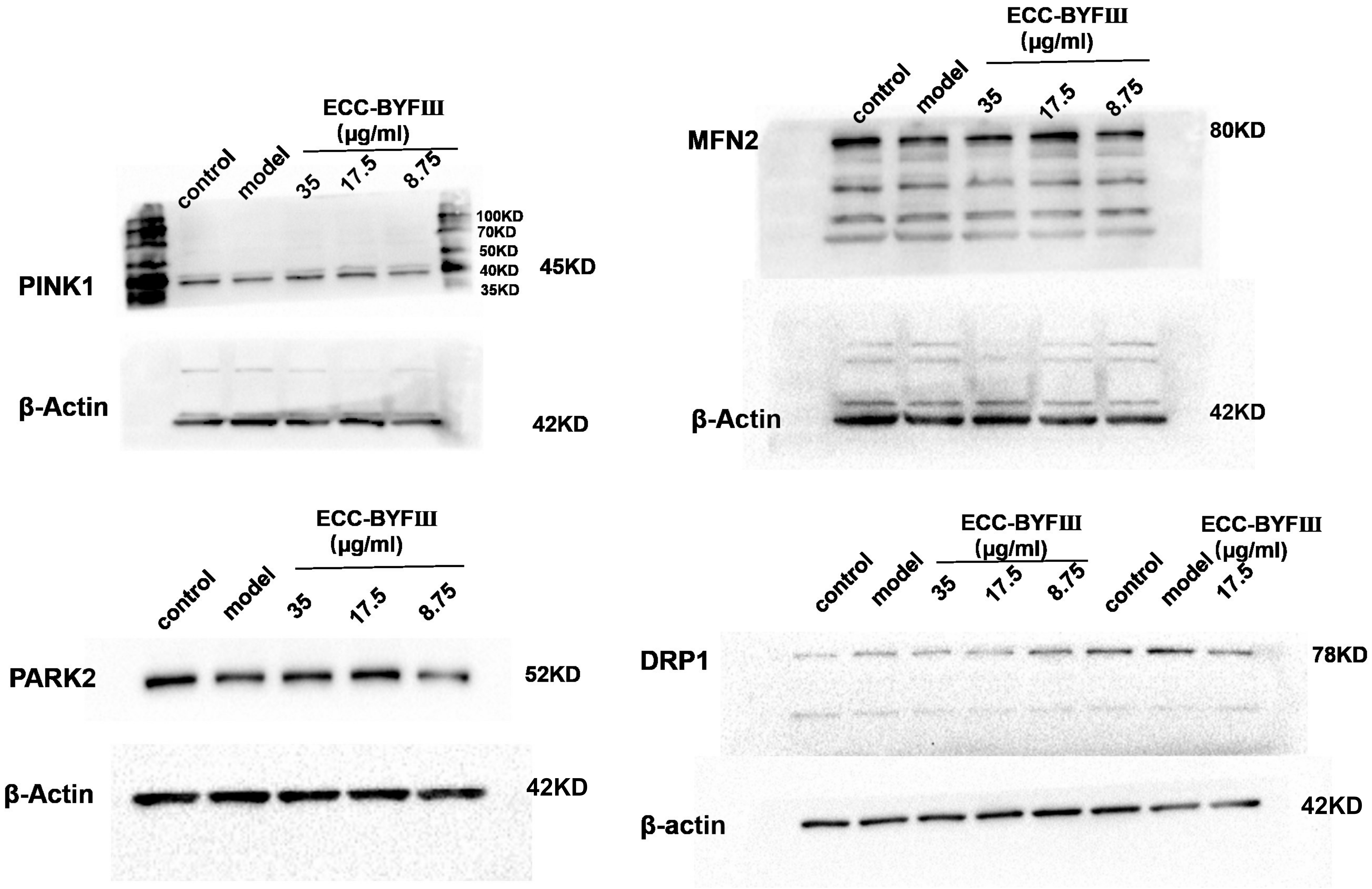

Supplementary Figure 2

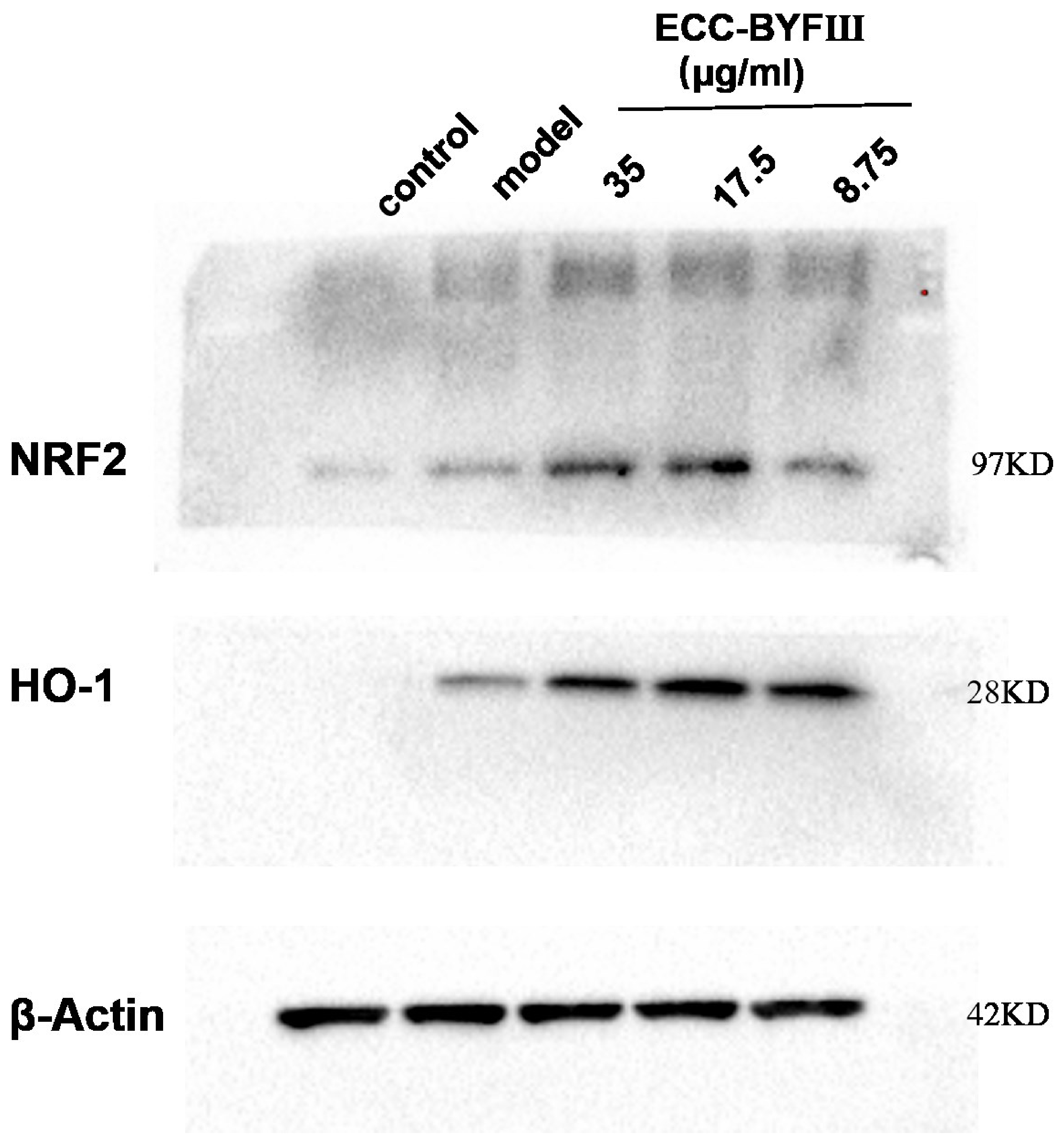

Supplementary Figure 3

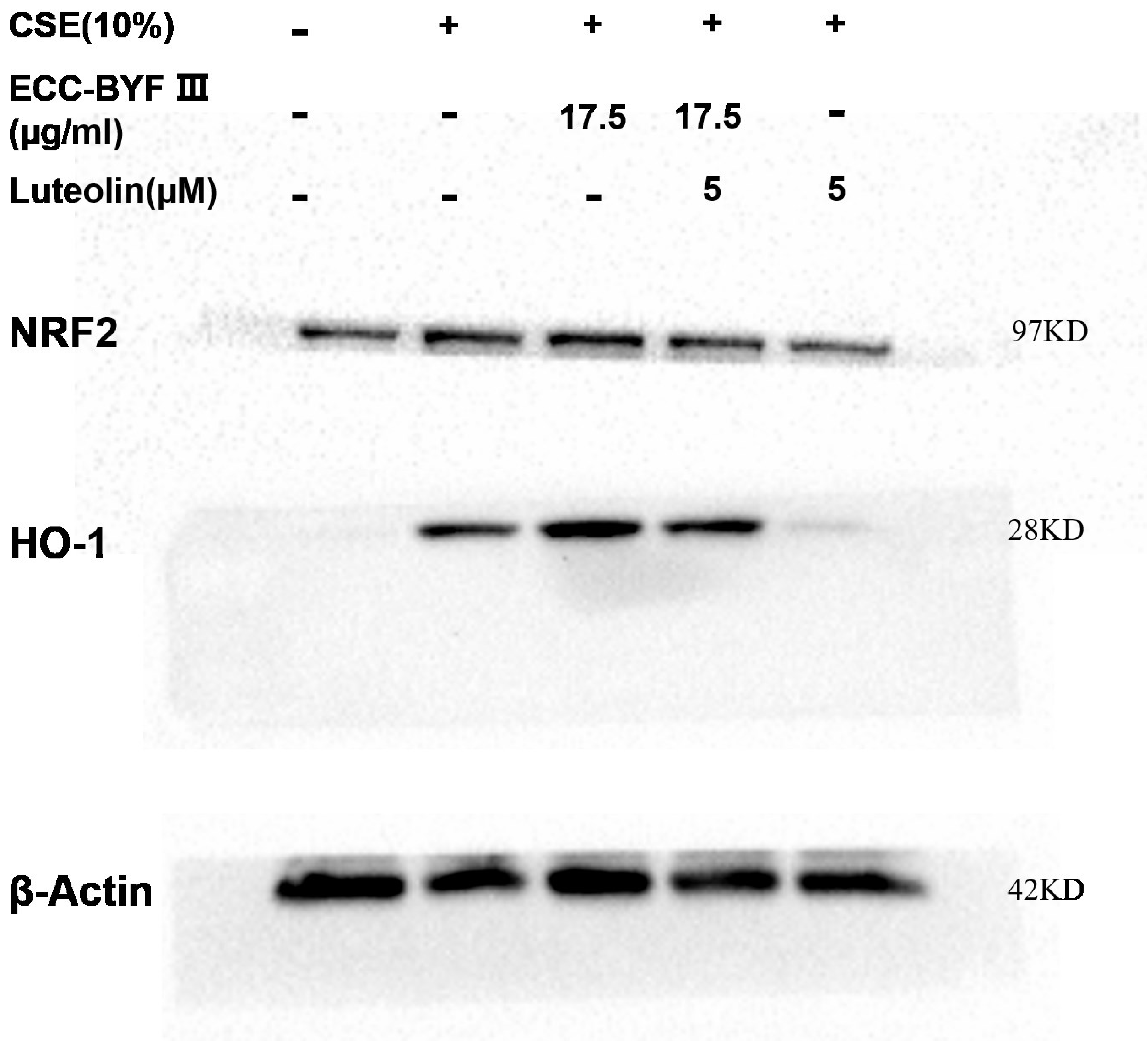

Supplementary Figure 4

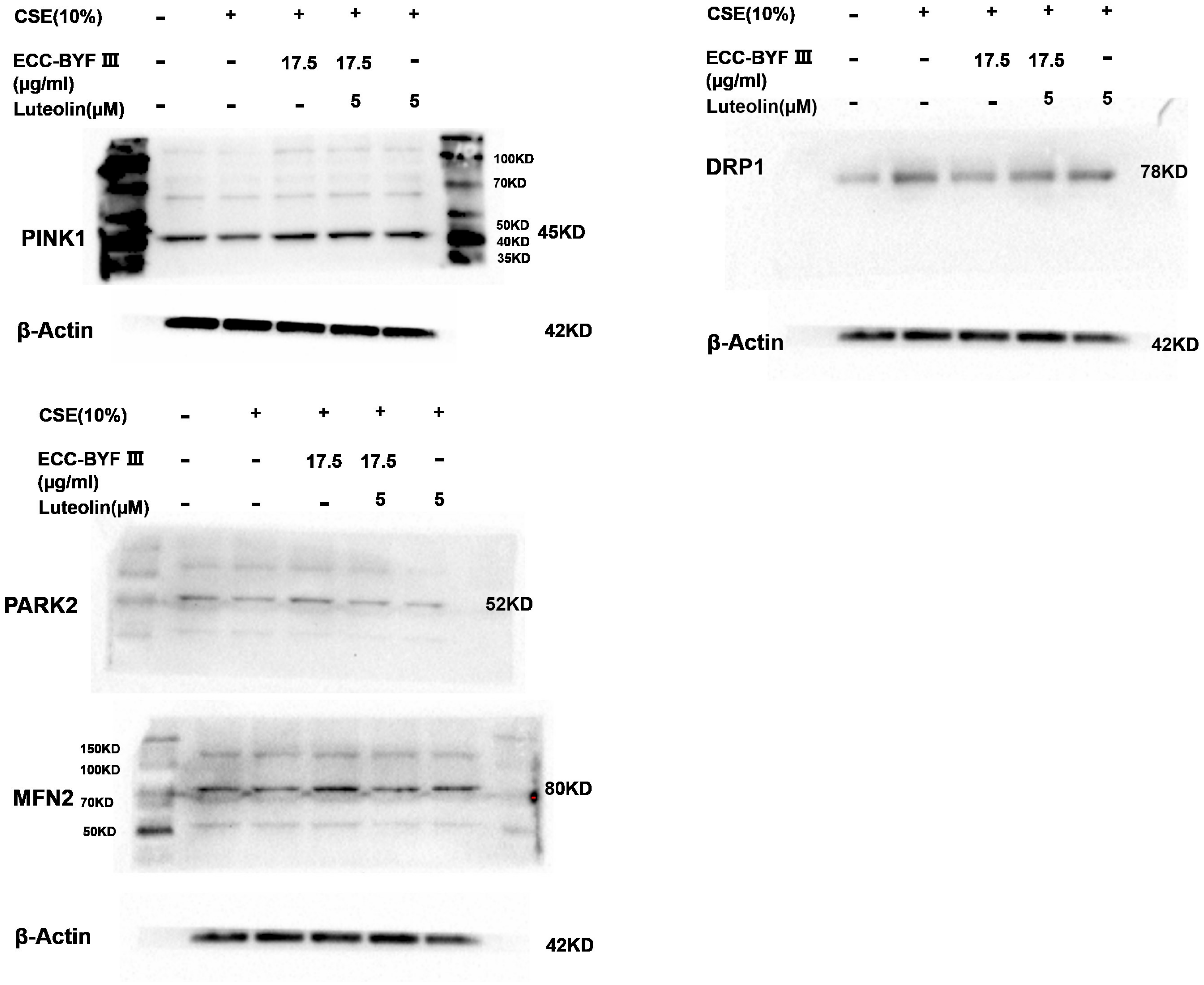

Supplementary Figure 5

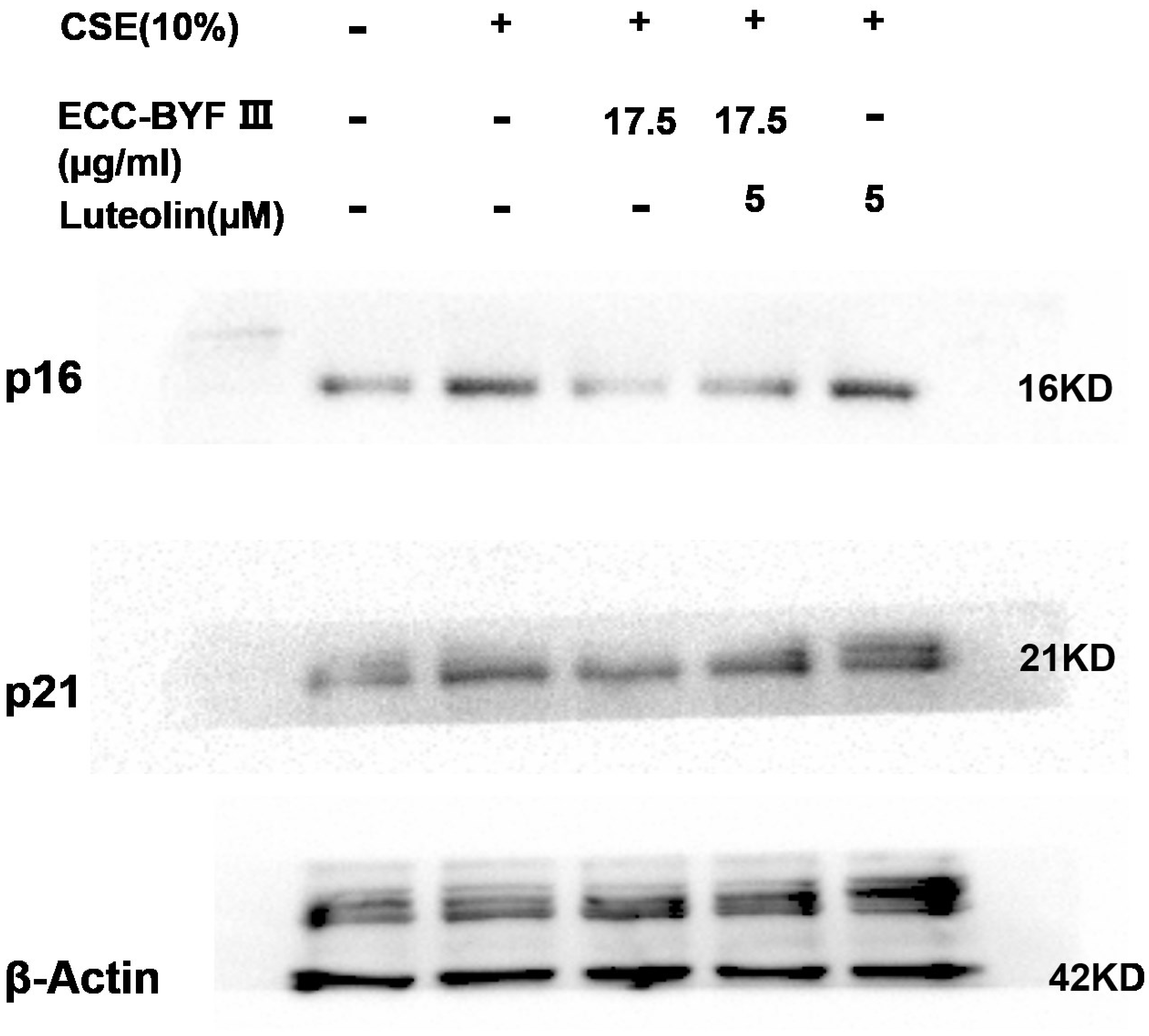

Supplementary Figure 6
